# The Response of the Rodent Gut Microbiome to Broad-Spectrum Antibiotics Is Different in Males and Females

**DOI:** 10.3389/fmicb.2022.897283

**Published:** 2022-06-09

**Authors:** Gonzalo Parodi, Gabriela Leite, Maya L. Pimentel, Gillian M. Barlow, Alyson Fiorentino, Walter Morales, Mark Pimentel, Stacy Weitsman, Ruchi Mathur

**Affiliations:** ^1^Medically Associated Science and Technology (MAST) Program, Cedars-Sinai, Los Angeles, CA, United States; ^2^Karsh Division of Gastroenterology and Hepatology, Department of Medicine, Cedars-Sinai, Los Angeles, CA, United States; ^3^Division of Endocrinology, Diabetes, and Metabolism, Department of Medicine, Cedars-Sinai, Los Angeles, CA, United States

**Keywords:** antibiotics response, antibiotics recovery, small bowel microbiome, stool microbiome, sex, male, female

## Abstract

Gut microbiome composition is different in males and females, but sex is rarely considered when prescribing antibiotics, and sex-based differences in gut microbiome recovery following antibiotic treatment are poorly understood. Here, we compared the effects of broad-spectrum antibiotics on both the stool and small bowel microbiomes in male and female rats. Adult male and female Sprague Dawley rats were exposed to a multi-drug antibiotic cocktail for 8 days, or remained unexposed as controls. Following cessation of antibiotics, rats were monitored for an additional 13-day recovery period prior to euthanasia. Baseline stool microbiome composition was similar in males and females. By antibiotic exposure day 8 (AbxD8), exposed male rats exhibited greater loss of stool microbial diversity compared to exposed females, and the relative abundance (RA) of numerous taxa were significantly different in exposed males vs. exposed females. Specifically, RA of phylum Proteobacteria and genera *Lactobacillus*, *Sutterella*, *Akkermansia*, and *Serratia* were higher in exposed males vs. exposed females, whereas RA of phyla Firmicutes and Actinobacteria and genera *Turicibacter* and *Enterococcus* were lower. By 13 days post antibiotics cessation (PAbxD13), the stool RA of these and other taxa remained significantly different from baseline, and also remained significantly different between exposed males and exposed females. RA of phyla Firmicutes and Actinobacteria and genus *Enterococcus* remained lower in exposed males vs. exposed females, and genus *Sutterella* remained higher. However, RA of phylum Proteobacteria and genus *Akkermansia* were now also lower in exposed males vs. females, whereas RA of phylum Bacteroidetes and genus *Turicibacter* were now higher in exposed males. Further, the small bowel microbiome of exposed rats on PAbxD13 was also significantly different from unexposed controls, with higher RA of Firmicutes, *Turicibacter* and *Parabacteroides* in exposed males vs. females, and lower RA of Bacteroidetes, Proteobacteria, Actinobacteria, *Oscillospira*, *Sutterella*, and *Akkermansia* in exposed males vs. females. These findings indicate that broad-spectrum antibiotics have significant and sex-specific effects on gut microbial populations in both stool and the small bowel, and that the recovery of gut microbial populations following exposure to broad-spectrum antibiotics also differs between sexes. These findings may have clinical implications for the way antibiotics are prescribed.

## Introduction

Antibiotics represent one of the twentieth century’s most important medical discoveries and have saved millions of lives over the last 100 years (Moser et al., 2019). However, concerns have arisen regarding the overuse of antibiotics, which can lead to microbial resistance (Centers for Disease Control and Prevention, 2020) and other problems (Cox and Blaser, 2015). An estimated 249.8 million antibiotics were prescribed to outpatients in the United States in 2018 (Centers for Disease Control and Prevention, 2020), but it has been suggested that up to one third of these prescriptions may have been unnecessary (Centers for Disease Control and Prevention, 2019). Antibiotics are also known to affect the gut microbiome, and both early and repeated exposures to antibiotics during childhood have been associated with long-term sequelae such as the development of obesity (Cox and Blaser, 2015; Turta and Rautava, 2016; Vallianou et al., 2021) and the acceleration of autoimmunity leading to Type 1 diabetes (T1D; Fuhri Snethlage et al., 2021). Whether these associations are directly linked to antibiotic exposure or result from antibiotic perturbation of gut microbial composition, remains unclear.

There are also sex differences in the composition of the gut microbiome, termed the “microgenderome” (Flak et al., 2013; Markle et al., 2013), which may affect immune responses (Flak et al., 2013; Markle et al., 2013; Markle and Fish, 2014; Rizzetto et al., 2018), how the gut microbiota respond to, and even regulate, steroid sex hormones (Rizzetto et al., 2018), and susceptibility to disease (Cooper and Stroehla, 2003; Trivedi and Hirschfield, 2021). Studies in mouse models indicate that T1D is more prevalent in female non-obese diabetic (NOD) mice when compared to males, but these sex differences are absent in germ-free mice, which lack gut microbes (Markle et al., 2013). Further, transferring cecal microbiota from male NOD mice into females protects against T1D development (Markle et al., 2013), and this finding was linked to increased testosterone levels, consistent with other data suggesting that testosterone can have protective effects in autoimmune diseases, which typically have greater prevalence or severity in women (Markle and Fish, 2014). In addition to these endogenous sex-based differences in male and female gut microbial composition, antibiotic treatments appear to also have sex-specific effects on the gut microbiome (Whitley and Lindsey, 2009; Markle et al., 2013; Harrison et al., 2019). In humans, studies exploring the effects of antibiotic administration during childhood also found that these effects appeared to differ between the sexes, with boys being more likely than girls to be overweight or have a higher body mass index (BMI) following early and/or repeated exposure to antibiotics, and that this was particularly true for broad-spectrum antibiotics, which have greater impacts on the gut microbiome (Azad et al., 2014; Murphy et al., 2014; Saari et al., 2015). Studies in mice have also shown sex-specific differences in the effects of antibiotics on the gut microbiome (Gao et al., 2019; Harrison et al., 2019) and the production of microbial metabolites including short chain fatty acids (SCFAs; Gao et al., 2019).

Despite these findings, sex is not currently considered in the “National Action Plan for Combating Antibiotic-Resistant Bacteria: 2020–2025,” which is intended to improve and develop the best practices for antibiotic use (Federal Task Force on Combating Antibiotic-Resistant Bacteria, 2020). Moreover, while the above-described studies have identified sex-specific effects of antibiotics on the gut microbiome (Gao et al., 2019; Harrison et al., 2019), sex-based differences in the recovery of the gut microbiome following antibiotic treatment have not been adequately explored. Lastly, these studies and the majority of other gut microbiome studies rely on stool or cecal samples as surrogates for the entire gut microbiome. However, the small bowel is primarily responsible for digestion and the absorption of nutrients and also plays key roles in immune responses and in maintaining gut barrier integrity (Ko and Chang, 2015; Rios et al., 2016; Burgueno and Abreu, 2020). We have previously shown that the human stool and small bowel microbiomes are significantly different from each other (Leite et al., 2020), such that stool findings cannot be used as a surrogate for small bowel microbial populations.

In this study, we sought to explore sex-specific changes in gut microbiome composition both in response to, and during recovery from, a broad-spectrum antibiotic cocktail in male and female Sprague Dawley rats. This cocktail, which included vancomycin, ampicillin, neomycin, and metronidazole, was chosen because it has previously been used to reduce endogenous gut microbial levels prior to the transfer of cecal contents (Fujisaka et al., 2016) and offered a broad-spectrum impact on a wide range of gut microbiota, as vancomycin affects Gram-positive bacteria (Antonoplis et al., 2019), ampicillin and neomycin are broad-spectrum antibiotics that affect both Gram-positive and Gram-negative bacteria (Kaushik et al., 2014; Sorkhy and Ghemrawi, 2020), and metronidazole affects both Gram-positive anaerobic and Gram-negative anaerobic bacteria (Löfmark et al., 2010). To determine whether there were different effects on the small bowel and stool microbiomes, microbial populations in stool samples and small bowel luminal contents from both male and female antibiotic-treated and control rats were analyzed and compared.

## Materials and Methods

### Rats

The study protocol was reviewed and approved by the Institutional Animal Care and Use Committee at Cedars-Sinai Medical Center (IACUC #8107). A total of 48 Sprague Dawley (SD) rats (Envigo, Somerset, NJ) were included in the study. Rats were obtained at 8 weeks of age and housed in pairs in a temperature-controlled room with 12 h light/12 h dark cycles and had *ad libitum* access to food (D11112201, OpenStandard Diet with 15 kcal%, Research Diets, Inc., New Brunswick, NJ) and water. Rats were allowed to acclimate for a period of 1 week, after which they were randomized into two groups to either receive a multi-drug antibiotic cocktail or remain unexposed as controls. The antibiotic exposed group (*N* = 31) included 16 males (M) and 15 females (F), and the control group (*N* = 17) included nine males and eight females. After cessation of antibiotics, the rats were monitored for an additional 13-day recovery period, after which all rats were euthanized (Supplementary Figure 1).

The rats were weighed throughout the study, and stool and serum samples were collected from the control and exposed groups prior to starting antibiotics (day 0/baseline), on the last day of antibiotic exposure [day 8 (ABXD8)], and on the day of euthanasia [13 days after cessation of antibiotics (PAbxD13); Supplementary Figure 1]. Stool and blood sample collection from control rats and exposed rats were performed in separate rooms to avoid cross-contamination.

### Antibiotic Cocktail

The exposed group received 0.5 g/L vancomycin (Sigma Aldrich, St. Louis, MO), 1 g/L ampicillin (Sigma Aldrich), 1 g/L neomycin (Sigma Aldrich), and 1 g/L metronidazole (Sigma Aldrich) *via* their drinking water for 8 days and then returned to normal drinking water for another 13 days. The control group received normal drinking water throughout the study.

### Stool and Blood Sample Collection

Stool samples were collected *via* stimulation of the anus during manual restraint. To avoid contamination of stool samples, two stool pellets were obtained from each rat. The first pellet was discarded, and the second pellet was collected directly from the anus into a 2 ml sterile tube and stored at −80°C for subsequent DNA extraction.

Blood samples were collected *via* tail-vein bleed and were allowed to coagulate for at least 30 min, then centrifuged at 6,500 rpm for 10 min at 4°C to separate serum. Serum was then aliquoted and stored at −80°C.

### Small Bowel Luminal Contents

For each rat at euthanasia (on PabxD13), a 5 cm piece of small bowel was harvested 15 cm from the cecum. This piece was placed in 4 ml of sterile 1X PBS and vortexed for 3 min, after which the tissue was removed and discarded. The liquid was then spun for 10 min at full speed (>13,000 rpm), the supernatant was discarded, and the resulting pellet was stored at −80°C for subsequent DNA extraction.

### Measurement of Blood Urea Nitrogen and Creatinine Levels

Circulating blood urea nitrogen (BUN) and creatinine levels were measured in serum samples using Urea/BUN and CREAJ Gen.2 kits (Roche Diagnostics, Indianapolis, IN, Cat. 04460715190 and Cat. 04810716190 respectively) on a COBAS integra 400 Plus System (Roche Diagnostics).

### Stool DNA Extraction and Isolation

Approximately 0.1 g of each stool sample was homogenized in 200 ml of sterile 1X PBS in a 2 ml sterile tube, and microbial DNA was isolated from each homogenate using the MagAttract PowerSoil DNA KF Kit (Qiagen, Hilden, Germany) following the manufacturer’s protocol with a few modifications. The lysis step was carried out by adding garnet beads and lysis buffer to each sample, followed by mechanical disruption for 10 min and high-speed centrifugation for 10 min. DNA was purified from the resulting supernatant on a KingFisher Duo (Thermo Fisher Scientific, Waltham, MA) following the manufacturer’s protocol. DNAs were quantified on a NanoDrop One™ Spectrophotometer (Thermo Fisher Scientific) and diluted to a concentration of 5 ng/ml for use in 16S rRNA sequencing.

### Library Preparation and 16S rRNA Sequencing and Analysis

Specific primers (S-D-Bact-0341-b-S-17 and S-D-Bact-0785-a-A-21; Klindworth et al., 2013) modified to include Illumina sequencing adapters (Leite et al., 2019) were used to amplify the 16S V3 and V4 regions as recommended by Illumina.^1^ An Agilent 2100 Bioanalyzer System was used to assess library quality. Paired-end sequencing of amplicons (2 × 301 cycles) was performed on a MiSeq system (Illumina, San Diego, CA) using the MiSeq reagent v3 Kit, with 600 cycles and 10%–15% PhiX (Illumina).

### Sequencing and Statistical Analysis

The Operational Taxonomic Unit (OTU) clustering tool available at CLC Genomics Workbench v.10.1.1 and CLC Microbial Genomics Module v.2.5 (Qiagen) software were used to perform reference-based OTU clustering and taxonomic analyses against the Greengenes Database 2013 with 97% similarity (Leite et al., 2019). Default parameters were used for minimum occurrences and chimera crossover cost, and the creation of new OTUs was not allowed. Low depth samples (<5,000 sequences per sample) were excluded from the analysis, and microbial alpha diversity indices were calculated. Bray–Curtis metric was used to calculate inter-sample variability (beta diversity).

Core microbiome analysis and correlation constructions were performed using the Core Microbiome tool and correlation network tool available at MicrobiomeAnalyst (Dhariwal et al., 2017; Chong et al., 2020). The core microbiome at phylum level was generated considering taxa present in at least 60% of samples.

Predictions for significant differentially abundant OTUs between different groups were performed using a rarefied OTU table. Multiple comparisons and statistical analyses were performed using CLC Genomics Workbench v. 10.1.1, CLC Microbial Genomics Module v. 2.5 (Qiagen), and MicrobiomeAnalyst (Dhariwal et al., 2017; Chong et al., 2020). The OTU table was rarefied to the minimal number of reads assigned to a sample, and a Negative Binomial GLM model was used to obtain maximum likelihood estimates for the fold change (FC) of an OTU between different groups. The Wald test was used for determination of significance, and *p* values were corrected using False Discovery Rate (FDR).

Two-tailed Spearman’s correlations, statistical tests and graph construction were carried out with rarefied OTU tables using GraphPad Prism 7.02 (GraphPad Software, La Jolla, CA; Weiss et al., 2016) and IBM SPSS Statistics Version 24. IBM SPSS Statistics Version 24 was also used for statistical comparisons of weight and serum biomarkers between groups.

### Microbial Pathway Prediction Analysis

PICRUSt-inferred functional analysis of microbial communities in stool and the small bowel was performed and visualized using BURRITO (McNally et al., 2018). Differences in mean abundance between female and male rats on AbxD8 and PAbxD13 were determined using Wilcoxon rank-sum test, and the *p* values were adjusted based on Benjamini-Hochberg FDR method.

## Results

### Antibiotic Exposure Induces More Sustained Weight Loss in Male vs. Female Rats

**Figure 1:**
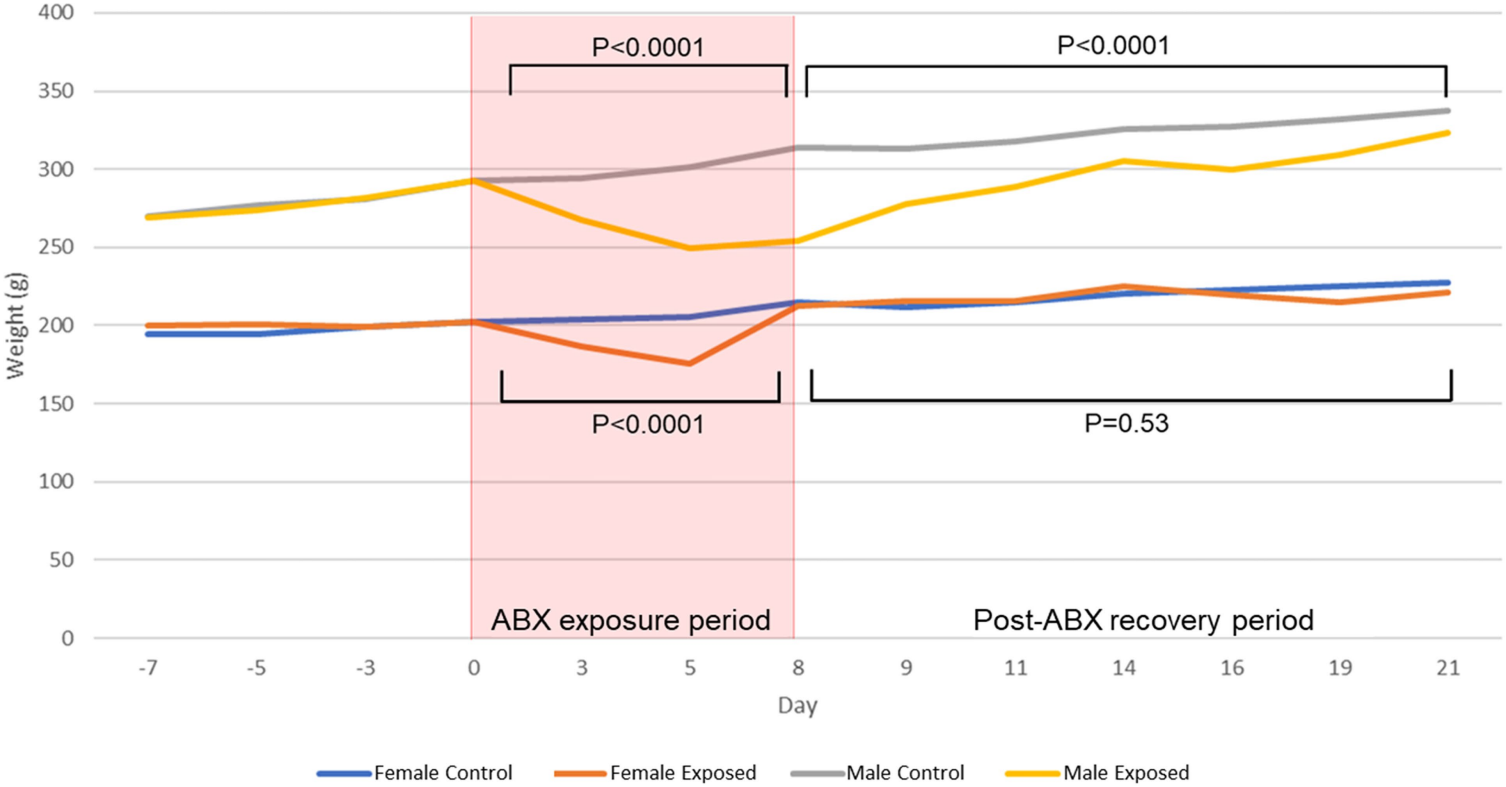
Average weights in male and female control and antibiotic-exposed rats at baseline, during the 8-day antibiotic exposure period, and during the 13-day recovery period following cessation of antibiotics. *P* values denote differences between antibiotic-exposed rats and controls of the same sex during the antibiotic-exposure and recovery periods.

After a 1-week acclimation period, male and female Sprague–Dawley rats (*N* = 48) were randomized to either receive a multi-drug antibiotic cocktail (*N* = 31) or remain unexposed as controls (*N* = 17; Supplementary Figure 1). At baseline (day 0), average weights were similar in all male (M) and female (F) rats (*p* = 0.97 and *p* = 0.78, respectively; Figure 1; Supplementary Table 1). During the 8-day antibiotic exposure period, both exposed males (*N* = 16) and exposed females (*N* = 15) lost significant amounts of weight (*p* < 0.0001; Figure 1). The greatest difference in weights between exposed and control rats was on the 5th day of antibiotic exposure, for both males (*p* < 0.0001) and females (*p* < 0.0001; Figure 1; Supplementary Table 1). To determine whether dehydration might contribute to this weight loss, BUN-to-creatinine ratio (BCR) was measured. Both male and female exposed rats had significant increases in BCR from baseline to day 8 of antibiotic exposure (AbxD8; *p* < 0.0001), consistent with the weight loss observed during this time period (Supplementary Table 1; Supplementary Figure 2).

By the end of the study, i.e., at 13 days post cessation of antibiotics (PAbxD13), the weights of female exposed rats were no longer significantly different from those of controls (*p* = 0.53; Figure 1). In contrast, the weights of male exposed rats did not fully recover and remained significantly lower than controls throughout the 13-day recovery period (*p* < 0.0001; Figure 1). On PAbxD13, BCRs in exposed males had decreased significantly when compared to AbxD8, and were now lower than in male controls (*p* = 0.007; Supplementary Table 1; Supplementary Figure 2A). BCRs in exposed females on PAbxD13 had also decreased significantly when compared to AbxD8 and were not significantly different when compared to controls (*p* = 0.143; Supplementary Table 1; Supplementary Figure 2B).

### Effects of Antibiotic Exposure on Total Stool DNA Levels in Male and Female Rats

Total stool DNA levels were significantly decreased on AbxD8 when compared to baseline in both exposed male (*p* = 0.0003) and exposed female (*p* < 0.0001) rats, suggesting a loss of microbial biomass, but returned to baseline levels by PAbxD13 in both males and females (*p* > 0.999; Supplementary Figure 3A). Despite similar total stool DNA levels at baseline (*p* = 0.7692), male exposed rats had more total stool DNA than female exposed rats on AbxD8 (*p* = 0.0001; Supplementary Figure 3B). By PAbxD13, there was no longer any significant difference in total stool DNA levels in exposed males and exposed females (*p* = 0.3791; Supplementary Figure 3B).

### Male Rats Exhibit Greater Loss of Stool Microbial Alpha and Beta Diversity During Antibiotic Exposure When Compared to Females

**Figure 2:**
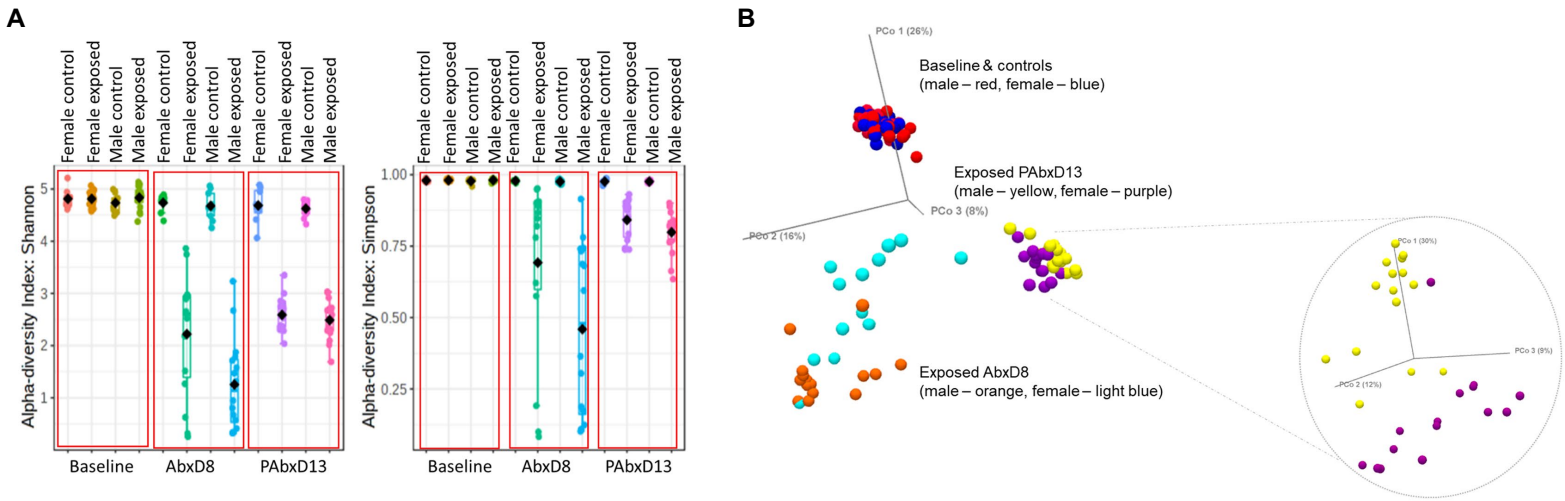
Microbial alpha and beta diversity in stool samples from male and female control and exposed rats. **(A)** Stool alpha diversity in male and female control and exposed rats at baseline, on AbxD8, and on PAbxD13, as measured by Shannon’s and Simpson’s indices. **(B)** Stool beta diversity in male and female exposed rats at baseline and all controls (males—red and females—blue), on AbxD8 (exposed males—orange and exposed females—light blue), and on PAbxD13 (exposed males yellow and exposed females purple).

Stool microbial alpha diversity was analyzed in samples from all rats at baseline, on AbxD8, and on PAbxD13, using two different indices: Shannon’s and Simpson’s. At baseline, there were no significant differences in alpha diversity between groups (*p* > 0.05; Table 1; Figure 2A). After 8 days of antibiotic exposure, both male and female exposed rats exhibited significantly lower alpha diversity when compared to baseline and to controls (*p* < 0.0001; Figure 2A), with even greater loss of alpha diversity in male exposed rats compared to exposed females (*p* = 0.01; Table 1; Figure 2A). On PAbxD13, microbial alpha diversity (Shannon’s and Simpson’s indices) had increased in both male and female exposed rats but remained significantly lower than in controls (*p* < 0.05; Figure 2A), and no statistical differences were observed between male and female exposed rats (*p* = 0.44; Table 1; Figure 2A).

**Table 1:** Overview of the most significant changes in taxa seen post antibiotic exposure in males compared to females at the phylum, family, and genus levels in both the stool and small bowel microbiomes.

	Male controls vs. female controls	Male ABX exposed vs. female ABX exposed	
**Stool microbiome**	**Baseline**	**AbxD8**	**PAbxD13**
**Alpha diversities**			
Simpson’s index	NS	↓	NS
Shannon’s index	NS	↓	NS
**Phylum level**			
Firmicutes^#^	NS	↓	↓
Bacteroidetes^#^	NS	NS	↑
Proteobacteria^#^	NS	↑	↓
Verrucomicrobia	↓	↑	↓
Actinobacteria	NS	↓	↓
**Family level**			
Verrucomicrobiaceae	↓	↑	↓
Unknown family, order RF32	↓	NS	NS
Corynebacteriaceae	↑^*^	NS	NS
Enterobacteriaceae	NS	↑^*^	↓
Staphylococcaceae	NS	NS	↑
**Genus level**			
*Lactobacillus*^#^	NS	↑	NS
*Oscillospira*^#^	NS	NS	NS
*Ruminococcus*^#^	NS	NS	↓
*Bacteroides*^#^	NS	NS	NS
*Sutterella*^#^	NS	↑	↑
*Turicibacter*^#^	NS	↓	↑
*Parabacteroides*^#^	NS	NS	↑
*Akkermansia*	↓	↑	↓
Unknown genus, order RF32	↓	NS	NS
*Lactococcus*	↓	NS	↓
*Coprococcus*	↓	NS	NS
Unknown genus, Enterobacteriaceae	NS	↑	↓
*Serratia*	NS	↑	NS
*Enterococcus*	NS	↓	↓
**Small bowel microbiome**	**PAbxD13**		**PAbxD13**
**Alpha diversities**			
Simpson’s index	NS		NS
Shannon’s index	NS		NS
**Phylum level**			
Firmicutes^#^	NS		↑
Bacteroidetes^#^	NS		↓
Proteobacteria	NS		↓
Verrucomicrobia	↓		↓
Actinobacteria	NS		↓
**Genus level**			
*Lactobacillus*	↑		NS
*Oscillospira*	NS		↓
*Ruminococcus*	NS		NS
*Bacteroides*	NS		NS
*Sutterella*	NS		↓
*Turicibacter*	NS		↑
*Parabacteroides*	NS		↑
*Akkermansia*	↓		↓
Unknown genus, order RF32	NS		NS
*Lactococcus*	NS		NS
*Coprococcus*	NS		↓

*NS, not significant; ↑, higher relative abundance in male rats compared to female rats; and ↓, lower relative abundance in male rats compared to female rats.*

* Indicates trend toward significance (*p* < 0.05, FDR *p* > 0.05).

# Taxa that were part of the core microbiome in control female and male rats.

Beta diversity analysis revealed that stool microbial profiles were similar in all rats at baseline (PERMANOVA *p* > 0.05), and that there were no significant differences between control males and control females at any timepoint during the study (PERMANOVA p > 0.05; Figure 2B). On AbxD8, the stool microbial profiles of exposed male and female rats were significantly different from baseline (PERMANOVA *p* = 0.00066 and *p* = 0.00066 respectively; Figure 2B) and also differed significantly from each other (PERMANOVA *p* = 0.00198; Figure 2B). On PAbxD13, the stool microbiomes of both male and female exposed rats had returned to profiles closer to, but still significantly different from, the profiles at baseline (PERMANOVA *p* = 0.00066 and *p* = 0.00066 respectively). Moreover, the profiles in male and female exposed rats remained significantly different from each other (PERMANOVA *p* = 0.00066; Figure 2B).

### Stool Core Microbial Profiles Are Similar in Male and Female Rats at Baseline

At baseline, the three most prevalent phyla in the core stool microbiome (defined as the most widespread microbial components of the microbiome found in all rats (Risely, 2020)) were the same in both male and female rats (mean prevalence in both: Firmicutes ~62%, Bacteroidetes ~34%, and Proteobacteria ~2%; Table 1; Supplementary Figure 4). The only difference found between male and female microbiomes at baseline was in phylum Verrucomicrobia, which was not part of the core stool microbiome (mean prevalence in both ~1%) and was significantly less prevalent in males vs. females [log2 fold change (FC) = −4.59, false discovery rate (FDR) *p* = 2.02E-6; Table 1; Supplementary Figure 4]. Interestingly, the prevalence of phylum Verrucomicrobia, primarily represented by genus *Akkermansia*, inversely correlated with weight (Spearman’s *R* = −0.361, *p* = 0.012), whereas the prevalences of phyla Firmicutes, Bacteroidetes, and Proteobacteria were not associated with weight (*p* > 0.05).

When analyzed at the family level, the baseline stool microbiomes of male and female rats were again highly similar. The relative abundances (RA) of only two families Verrucomicrobiaceae and an unknown family from order RF32 (phylum Proteobacteria) were lower in male vs. female rats (log2 FC = −4.88, FDR *p* = 8.35E−7; log2 FC = -1.60, FDR *p* = 7.66E−3, respectively; Table 1; Supplementary Figure 4). In addition, the RA of family Corynebacteriaceae (phylum Actinobacteria) trended toward being higher in male vs. female rats, although this did not reach significance after correction for FDR (log2 FC = 2.38, *p* = 0.02, FDR *p* > 0.05; Table 1; Supplementary Figure 4).

Further analysis at the genus level confirmed highly similar baseline stool microbiomes in male and female rats, with more than 100 shared features independent of sex. The most prevalent genera in both sexes were *Lactobacillus*, *Oscillospira* and *Ruminococcus* (phylum Firmicutes), *Bacteroides* (phylum Bacteroidetes), and *Sutterella* (phylum Proteobacteria), and the prevalence of non-assigned genera was equally distributed in both sexes (Table 1; Supplementary Figure 4). Only four genera had significantly lowered RA in males vs. females at baseline: *Akkermansia* (phylum Verrucomicrobia; log2 FC = −4.91, FDR *p* = 3.06E−6), consistent with published data (Gao et al., 2021), an unknown genus from order RF32 (phylum Proteobacteria; log2 FC = −1.80, FDR *p* = 5.95E−3), *Lactococcus* (phylum Firmicutes; log2 FC = -1.15, FDR *p* = 0.01), and *Coprococcus* (phylum Firmicutes; log2 FC = −1.39, FDR *p* = 0.029; Table 1; Supplementary Figure 4).

### Antibiotic-Induced Changes in the Stool Microbiome Differ in Male and Female Rats

**Figure 3:**
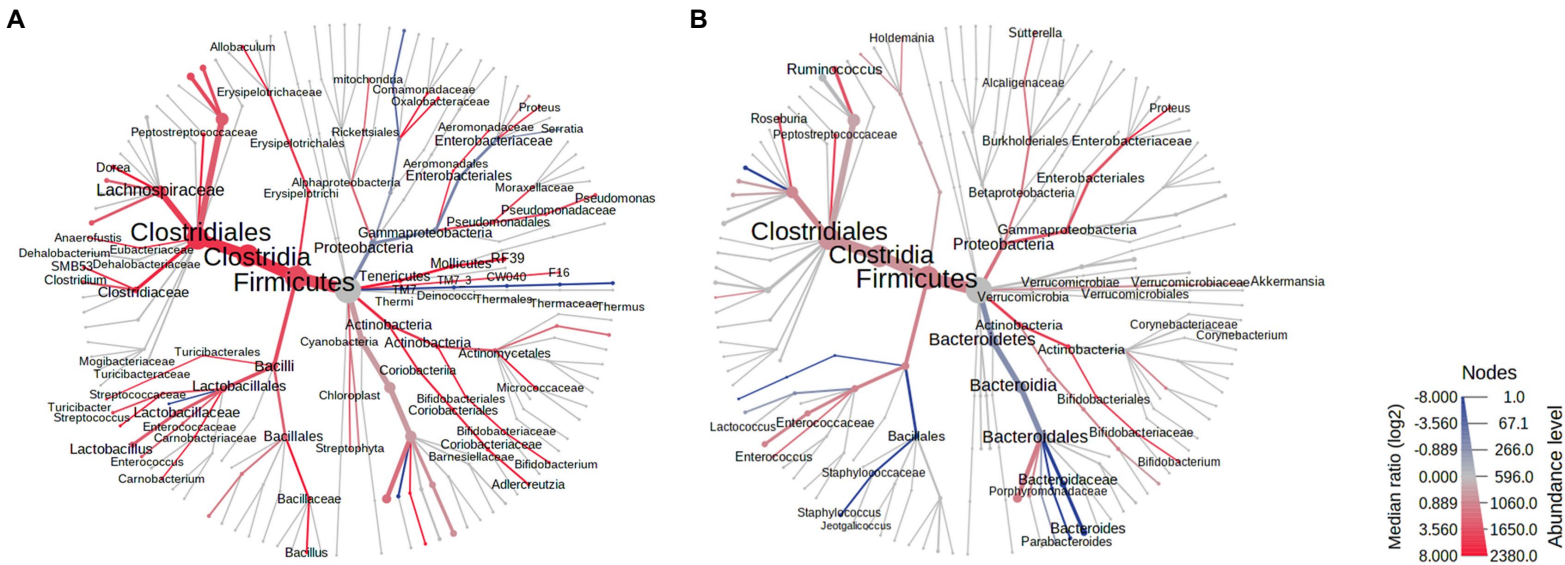
Heat trees of the stool microbiomes in male vs. female antibiotic-exposed rats. **(A)** Exposed males vs. exposed females on AbxD8. **(B)** Exposed males vs. exposed females on PAbxD13. Taxa with lower RA in males vs. females are shown in red. Taxa with higher RA in males vs. females are shown in blue. Nodes represent taxonomic levels. Greater line thicknesses and font sizes denote higher RA.

On AbxD8, the RA of two major phyla from the core stool microbiome were found to be significantly different in male vs. female exposed rats (Figure 3A). The RA of phylum Proteobacteria was significantly higher in male vs. female exposed rats (log2 FC = 1.80, FDR *p* = 1.77E−3), and the RA of phylum Firmicutes was significantly lower in male vs. female exposed rats (log2 FC = −1.11, *p* = 0.006; Table 1; Figure 3A). In addition, phylum Bacteroidetes, which was the third most prevalent phylum in the core stool microbiome at baseline, was significantly affected by antibiotic exposure and was almost undetectable in both exposed males and exposed females (mean prevalence in both sexes: <1%). Interestingly, the RA of phylum Verrucomicrobia, which was not part of the core stool microbiome at baseline (and lower in males than females at baseline), was increased in exposed males rats on AbxD8, and was significantly higher in male vs. female exposed rats (log2 FC = 6.15, FDR *p* = 3.67E−10, Table 1). The RA of phylum Actinobacteria, which was also not part of the core stool microbiome baseline, was significantly lower in male vs. female exposed rats (*p* = 0.003, Table 1).

When analyzed at the family level, the RA of 26 families were significantly different in the stool microbiota of male vs. female exposed rats on AbxD8 (Figure 3A). The greatest difference was in the RA of family Alcaligenaceae (phylum Proteobacteria), which was significantly higher in male vs. female exposed rats (log2 FC = 7.82, FDR *p* < 0.0001), as were the RA of Verrucomicrobiaceae (log2 FC = 5.67, FDR *p* = 2.62E-8), Brucellaceae (phylum Proteobacteria; log 2 FC = 4.45, FDR *p* = 7.23E-5), and the Bacteroidetes families Porphyromonadaceae (log2 FC = 3.10, FDR *p* = 2.79E-4), Bacteroidaceae (log2 FC = 1.94, FDR *p* = 0.01), and Prevotellaceae (log2 FC = 2.08, FDR *p* = 0.03), among others (Figure 3A). The RA of family Enterobacteriaceae, a major representative of phylum Proteobacteria (prevalence ~94%), also trended toward being higher in the stool microbiome of male vs. female exposed rats, although this did not reach significance after correction for FDR (FC = 1.52, *p* = 0.029, FDR *p* > 0.05). In contrast, the RA of the Firmicutes families Peptostreptococcaceae (log2 FC = −3.08, FDR *p* = 2.06E−7), Clostridiaceae (log2 FC = −2.85, FDR *p* = 2.06E−7), Turicibacteraceae (log2 FC = −2.65, FDR = 1.97E−3), and Lactobacillaceae (log2 FC = −2.87, FDR *p* = 9.80E−7), among others, were significantly lower in male vs. female exposed rats after antibiotic exposure (Figure 3A). Thus, while the overall RA of these taxa were lower than at baseline, there were further differences in RA between the sexes following antibiotic exposure.

At the genus level, the RA of 42 genera (including some unknown genera) were significantly different in the stool microbiota of male vs. female exposed rats on AbxD8 (Figure 3A). The RA of *Akkermansia* (phylum Verrucomicrobia; log2 FC = 6.60, FDR *p* = 8.03E-11) was increased in exposed males compared to baseline and were now significantly higher than in exposed females (Table 1). In addition, the association between *Akkermansia* and weight was even stronger than at baseline (Spearman’s *R* = −0.521, *p* = 0.008). When compared to exposed females, exposed males also had higher RA of Gram-negative bacteria from phylum Proteobacteria, including the genus *Sutterella*, which was part of the core stool microbiome in controls (log2 FC = 7.51, FDR *p* < 0.0001), as well as unknown genus from family Enterobacteriaceae (log2 FC = 2.16, FDR *p* = 0.02), and the genus *Serratia* (log2 FC = 1.52, *p* = 0.036, FDR *p* > 0.05; Table 1; Figure 3A). The RA of other genera that were part of the core stool microbiome in controls were lower in male vs. female exposed rats, including the Firmicutes genera *Enterococcus* (log2 FC = −3.27, FDR *p* = 7.76E−5), SMB53 (log2 FC = −2.41, FDR *p* = 6.45E-5) and *Turicibacter* (log2 FC = -1.72, *p* = 0.0014, FDR *p* = 0.08; Table 1; Figure 3A).

### The Recovery of the Stool Microbiome Following Cessation of Antibiotics Is Different in Males and Females, and Profiles Remain Different From Baseline

Stool samples were analyzed in all rats on PAbxD13, in order to compare the recovery of the stool microbiome in male vs. female exposed rats. At the phylum level, the prevalence of Actinobacteria, which was slightly lower in male vs. female exposed rats on AbxD8 (Figure 3A), was even lower in male vs. female exposed rats at 13 days on PAbxD13 (log2 FC = −2.55, FDR *p* = 1.61E−5; Table 1; Figure 3B). The prevalence of Bacteroidetes (one of the phyla most affected by antibiotic exposure) in female exposed rats on PAbxD13 was similar to that at baseline (~35% vs. ~34%, respectively), whereas the prevalence of Bacteroidetes in male exposed rats on PAbxD13 was higher than at baseline (~58% vs. ~34%, respectively), and was significantly higher than in exposed females (*p* = 0.0005; Table 1; Figure 3B).

At the family level, the RA of several families which had been significantly different in the stool microbiota of exposed males and females on AbxD8, remained significantly different on PAbxD13, including Peptostreptococcaceae and Turicibacteraceae (phylum Firmicutes), and Porphyromonadaceae and Bacteroidaceae (phylum Bacteroidetes; Figure 3B). Interestingly, the RA of family Enterobacteriaceae (phylum Proteobacteria), which was slightly higher in the stool microbiome of male vs. female rats on AbxD8, was significantly higher in the stool of females vs. males on PAbxD13 (Figure 3B). In addition, the RA of family Staphylococcaceae (phylum Firmicutes), which had not been significantly different between male and female exposed rats on AbxD8, was higher in the stool of male vs. female exposed rats on PAbxD13 (Figure 3B).

There were also significant differences between the stool microbiota of male vs. female exposed rats at the genus level on PAbxD13 (Figure 3B). Two of the genera present in the core stool microbiome of all rats, *Parabacteroides* (phylum Bacteroidetes) and *Turicibacter* (phylum Firmicutes), had the highest RA in male vs. female exposed rats on PAbxD13 (log2 FC = 11.75, FDR *p* < 0.0001; log2 FC = 6.93, FDR *p* < 0.0001, respectively), and the RA of other genera, including the Firmicutes genera *Staphylococcus* (log2 FC = 6.39, FDR *p* = 6.96E-8) and *Lactococcus* (log2 FC = 5.45, FDR *p* = 2.50E-4), were also higher in male vs. female exposed rats at 13 days on PAbxD13 (Table 1; Figure 3B). An unknown genus from family Peptostreptococcaceae (phylum Firmicutes) had the lowest RA in male vs. female exposed rats on PAbxD13 (log2 FC = -3.48, FDR *p* = 4.27E-8), and the RA of other genera were also lower in male vs. female exposed rats on PAbxD13, including the Firmicutes genera *Roseburia* (log2 FC = −2.99, FDR *p* = 7.31E−5), *Ruminococcus* (log2 FC = −1.64, FDR *p* = 0.02) and unknown genera from families Christensenellaceae (log2 FC = −3.23, FDR *p* = 2.15E−4) and Lachnospiraceae (log2 FC = −1.41, FDR *p* = 0.01); genus *Bifidobacterium* (phylum Actinobacteria, log2 FC = −2.68, FDR p = 0.02); and an unknown genus from family Enterobacteriaceae (phylum Proteobacteria, log2 FC = −3.01 FDR *p* = 6.67E−4; Figure 3B). The RA of genus *Akkermansia* (phylum Verrucomicrobia) appeared to return to baseline levels in both sexes, such that it was lower in the stool microbiome of male vs. female exposed rats on PAbxD13 (log2 FC = −0.44, *p* = 0.006, FDR *p* > 0.05, Table 1).

### Changes in the Stool Microbiome Following Exposure to Antibiotics Result in Changes in Predicted Microbial Functional Metabolic Pathways

**Figure 4:**
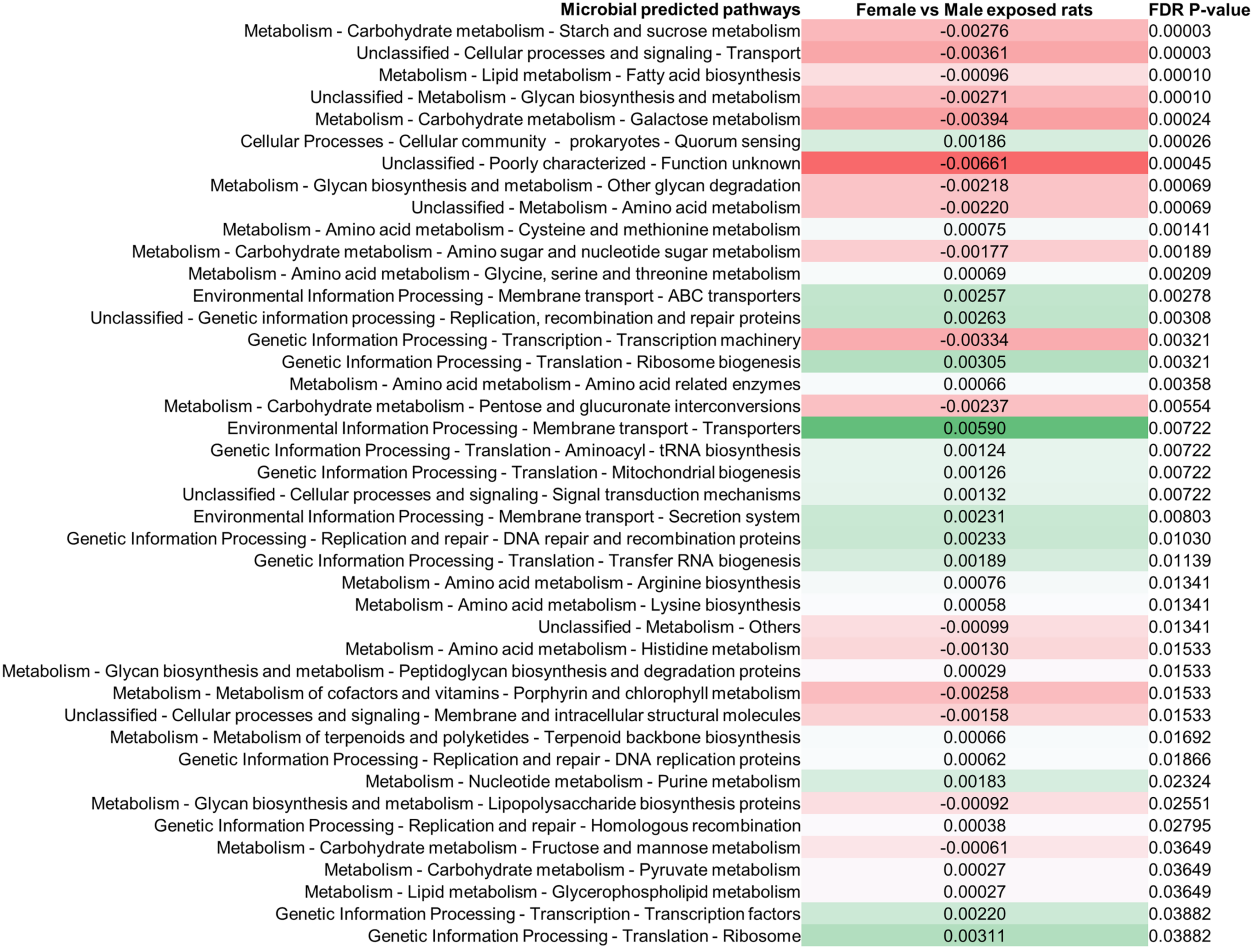
Differences in predicted stool microbial functional metabolic pathways in exposed male and female rats on PAbxD13. Pathways shown in red are predicted to be enriched in exposed males, and pathways shown in green are predicted to be enriched in exposed females. Only significant differences are shown.

Differences in the stool microbiomes of male vs. female exposed rats on PAbxD13 were associated with differences in the predicted microbial functional metabolic pathways (Supplementary Figure 5). The top five pathways were predicted to be enriched in male vs. female exposed rats, including starch and sucrose metabolism (FDR *p* = 0.00003), transport (FDR p = 0.00003), fatty acid biosynthesis (FDR *p* = 0.0001), glycan biosynthesis and metabolism (FDR p = 0.0001), and galactose metabolism (FDR *p* = 0.00024; Figure 4). No significant differences in predicted microbial functional metabolic pathways were identified when male controls were compared to female controls (Supplementary Figure 6).

### Antibiotic-Induced Changes in the Small Bowel Microbiome Also Differ Between Male and Female Rats

**Figure 5:**
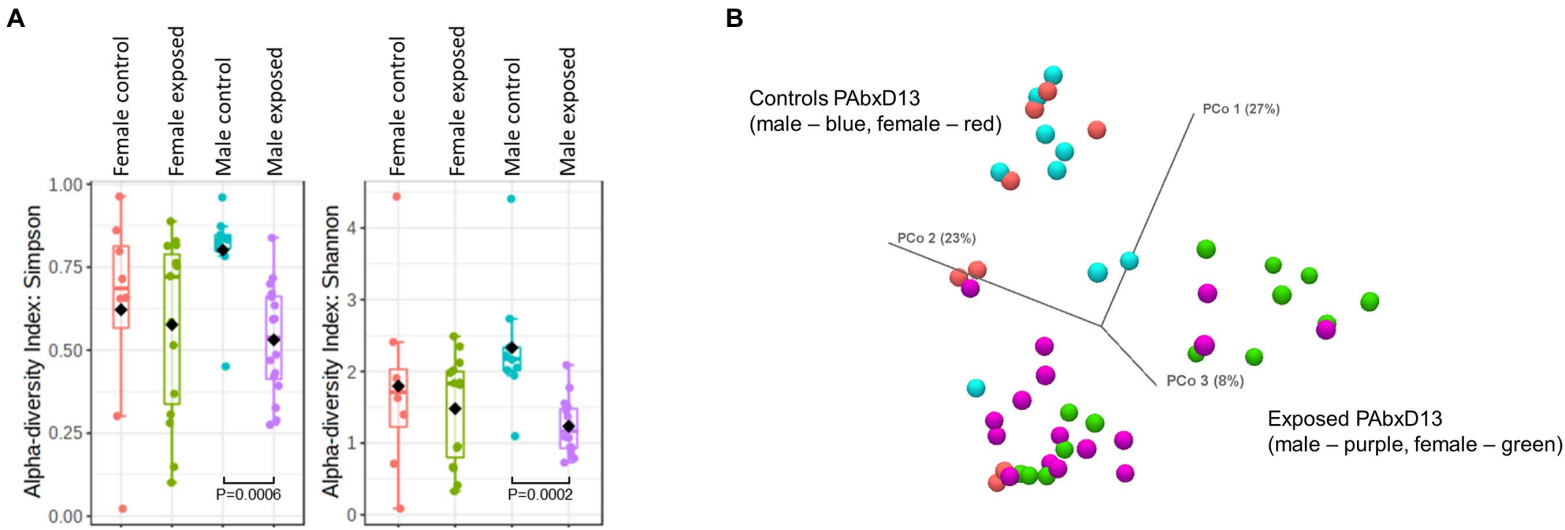
Microbial alpha and beta diversity in small bowel samples from male and female control and exposed rats. **(A)** Small bowel alpha diversity in male and female control and exposed rats on PAbxD13, as measured by Simpson’s and Shannon’s indices. Exposed males vs. control males: Simpson’s index *p* = 0.0006, Shannon’s index *p* = 0.0002. All other comparisons *p* = NS. **(B)** Small bowel beta diversity in male and female rats on PAbxD13 (control males—red, control females—blue, exposed males—purple, and exposed females—green). Male vs. female controls: PERMANOVA *p* = 1. Exposed females vs. exposed males PERMANOVA *p* = 0.01026. Exposed females vs. female controls: PERMANOVA *p* = 0.0022. Exposed males vs. male controls: PERMANOVA *p* = 0.00006.

Small bowel luminal contents were harvested from all rats on PAbxD13. Small bowel microbial alpha diversity appeared to be slightly higher in male control rats when compared to female controls (Simpson’s index *p* = 0.167, Shannon’s index *p* = 0.139, Figure 5A), and, similar to the stool microbiome, was significantly lower in exposed males vs. control males (Simpson’s index *p* = 0.0006, Shannon’s index *p* = 0.0002, Figure 5A). However, in contrast to the findings for stool, small bowel microbial alpha diversity was similar in exposed and control females (Simpson’s index *p* = 0.825, Shannon’s index *p* = 0.975, Figure 5A).

Beta diversity analysis revealed that, as for the stool microbiome, the small bowel microbial profiles in both male and female antibiotic-exposed rats were significantly different from those in non-exposed controls. Additionally, beta diversity analysis also revealed that the small bowel microbiome profiles in exposed females were significantly different from those in exposed males (PERMANOVA *p* = 0.01026), whereas those in male and female controls were similar (PERMANOVA *p* = 1; Figure 5B; Supplementary Figure 7). Interestingly, while small bowel alpha diversity was similar in control and exposed female rats, the microbiome profiles in these two groups were clearly distinct (PERMANOVA *p* = 0.0022, Figure 5B; Supplementary Figure 7), indicating that the microbial alpha diversity in female exposed rats was driven by taxa from different microbial communities.

**Figure 6:**
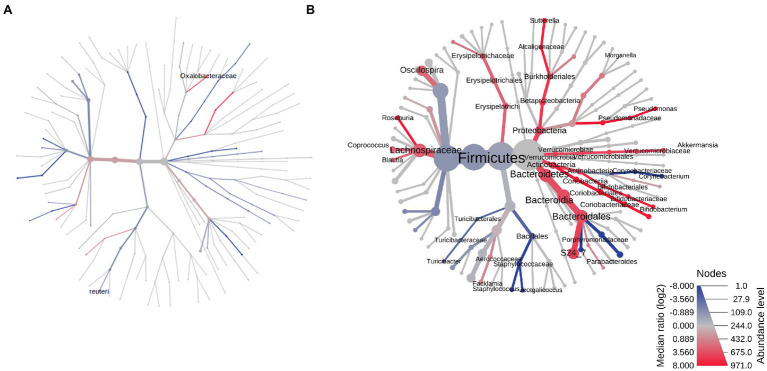
Heat trees of the small bowel microbiomes of male vs. female rats on PAbxD13. **(A)** Control males vs. control females. **(B)** Exposed males vs. exposed females. Taxa with lower RA in males vs. females are shown in red. Taxa with higher RA in males vs. females are shown in blue. Nodes represent taxonomic levels, and greater line thickness denotes higher RA.

The two most prevalent phyla in the small bowel microbiome at euthanasia were the same in both male and female control rats (mean prevalence in both groups: Firmicutes ~94% and Bacteroidetes ~5%) and, as for the stool microbiome, there were very few differences in the small bowel microbiome between male and female controls (Table 1; Figure 6A). The most significant difference was a higher RA of *Lactobacillus reuteri* (phylum Firmicutes) in male vs. female controls (FC = 1.44, *p* = 0.0078; Figure 6A). Similar to the stool microbiome, the RA of genus *Akkermansia* (phylum Verrucomicrobia) was significantly lower in the small bowel microbiome of male vs. female controls (Table 1, FC = −4.37, FDR *p* = 9.58E−3).

At euthanasia (on PAbxD13), differences in the small bowel microbiome in male vs. female exposed rats were even more profound than those identified in stool. The RA of five phyla were significantly different in exposed males vs. females, including Firmicutes (*p* = 0.00146), the most prevalent phylum in control rats, Actinobacteria (*p* = 0.00053), Verrucomicrobia (*p* = 0.0084), Proteobacteria (*p* = 0.0119), and Bacteroidetes (*p* = 0.0158; Table 1; Figure 6B). The direction of changes in RA of Firmicutes and Bacteroidetes in the small bowel in males vs. females following antibiotics exposure (Figure 6B) was different from that in stool (Table 1; Figure 3B). Specifically, the RA of phylum Firmicutes was 4.3-fold higher in the small bowel of male vs. female exposed rats (*p* = 0.00146), whereas the RA of phylum Bacteroidetes was 3.6-fold lower in exposed males vs. exposed females (*p* = 0.0158; Figure 6B). Although the overall changes in these phyla did not follow the direction seen in stool, many genera within these phyla showed similar changes in the stool and small bowel of male vs. female exposed rats, including the genera *Roseburia* (FC = −1.83, *p* = 0.0013) and *Staphylococcus* (phylum Firmicutes, FC = 8.02, *p* = 0.00044), and *Parabacteroides* (phylum Bacteroidetes, FC = 10.69, *p* = 0.01; Figure 6B).

In contrast, the direction of changes in the RA of phyla Actinobacteria (FC = −5.34), Verrucomicrobia (FC = −2.07), and Proteobacteria (FC = −2.88) in the small bowel microbiome (Figure 6B) was the same as in stool, i.e., the RA were lower in male vs. female exposed rats (Table 1; Figure 3B). Analysis at the genus level revealed lower RA of *Bifidobacterium* (phylum Actinobacteria, FC = −4.25, *p* = 0.00019), *Akkermansia* (phylum Verrucomicrobia, FC = −1.54, *p* = 0.0084) and *Sutterella* (phylum Proteobacteria, FC = −2.95, *p* = 0.015), among others, in male vs. female exposed rats (Table 1; Figure 6B).

### Changes in the Small Bowel Microbiome Following Exposure to Antibiotics Also Results in Changes in Predicted Microbial Functional Metabolic Pathways

**Figure 7:**
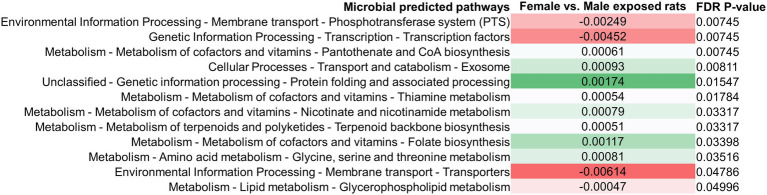
Differences in predicted small bowel microbial functional metabolic pathways in exposed male and female rats on PAbxD13. Pathways shown in red are predicted to be enriched in exposed males, and pathways shown in green are predicted to be enriched in exposed females. Only significant differences are shown.

Difference in the small bowel microbiomes of male vs. female exposed rats on PAbxD13 were also associated with differences in the predicted microbial functional metabolic pathways (Supplementary Figure 8). Within the top five pathways, two were predicted to be enriched in male vs. female exposed rats—phosphotransferase system (FDR *p* = 0.00745) and transcription factors (FDR *p* = 0.00745; Figure 7). Two pathways that were predicted to be enriched in the stool of female exposed rats when compared to exposed males—glycine, serine and threonine metabolism, and terpenoid backbone metabolism (Figure 4)—were also predicted to be enriched in the small bowel (FDR *p* = 0.035 and FDR *p* = 0.033, respectively, Figure 7). No significant differences were identified when male controls were compared to female controls (Supplementary Figure 9).

### The Small Bowel Microbiome and Stool Microbiome of Female Rats Have a Shared Microbial Signature After Antibiotic Exposure

As noted above, small bowel microbial alpha diversity was similar in female exposed and female control rats, but the two groups had distinct microbial profiles (Figure 5), suggesting the possibility that in female rats exposed to antibiotics, the microbial taxa normally found in controls were replaced with taxa from different microbial communities. To determine which microbial taxa drove these results, the microbial profiles in the small bowel were compared to those in stool.

**Figure 8:**
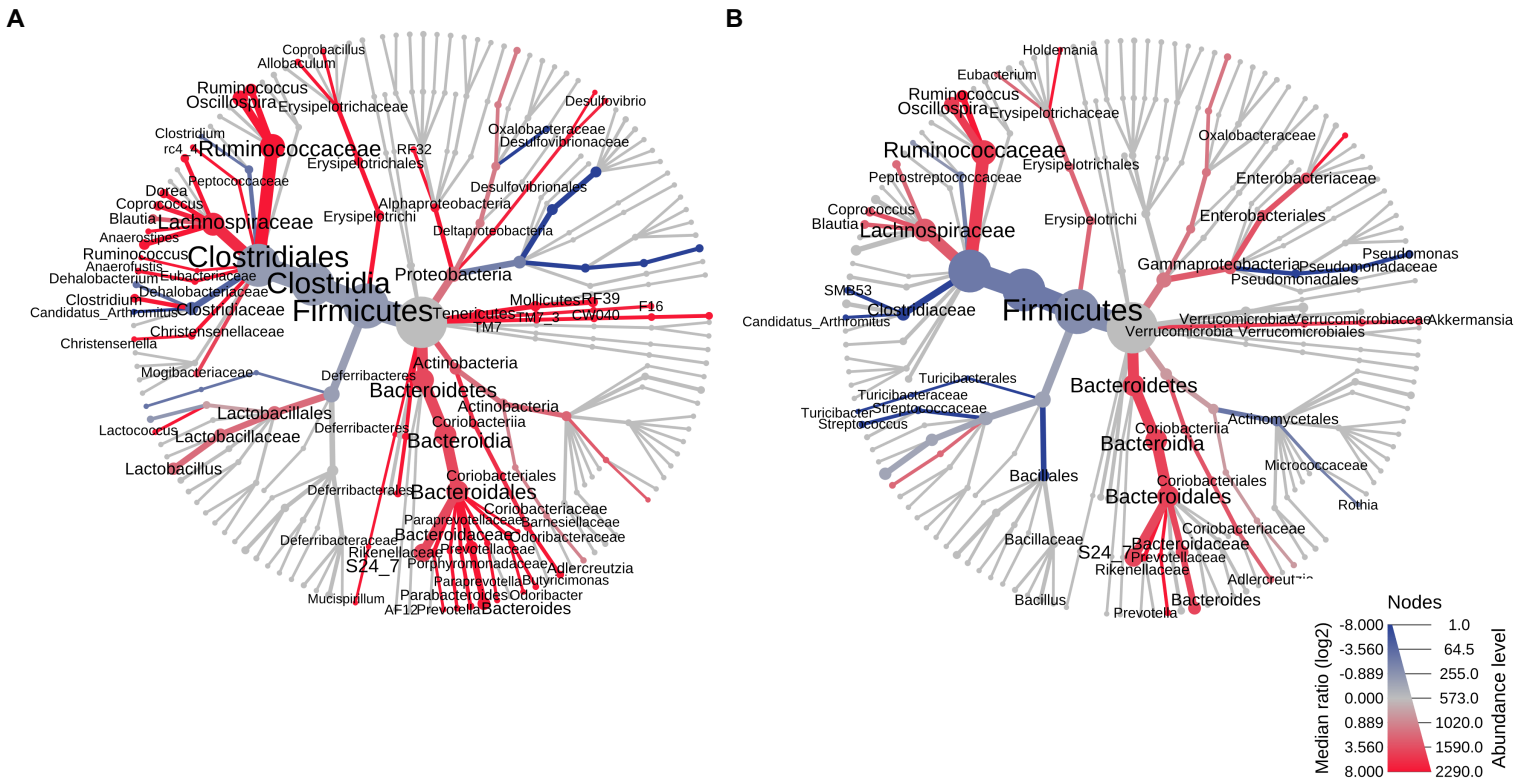
Heat trees of the stool vs. small bowel microbiomes in control and exposed female rats on PAbxD13. **(A)** Stool vs. small bowel microbiomes in control females. **(B)** Stool vs. small bowel microbiomes in exposed females. Taxa with lower RA in the small bowel vs. stool are shown in red. Taxa with higher RA in the small bowel vs. stool are shown in blue. Nodes represent taxonomic levels. Greater line thicknesses and greater font sizes denote higher RA.

The small bowel and stool microbiomes of female control rats exhibited some similarities, but also exhibited significant differences, including seven phyla, 10 classes, 10 orders, 23 families, and 28 genera (*p* < 0.05; Figure 8A). In female exposed rats, differences between the small bowel and stool microbiomes were less profound, and included three phyla, five classes, nine orders, 18 families, and 17 genera (p < 0.05; Figure 8B). Moreover, the taxa that were different in small bowel vs. stool in exposed female rats were not the same as those that were different in small bowel vs. stool in control females. Males exposed rats also exhibited differences between their stool and small bowel microbiomes, but the same taxa differed between stool and small bowel in both exposed rats and control males (Supplementary Figure 10).

Table 1 provides an overview of the most significant changes seen post antibiotic exposure in males and females at the phylum, family and genus levels, in both the stool and small bowel microbiomes.

## Discussion

In this study, we show that exposure to a broad-spectrum, multi-drug antibiotic cocktail has significant, sex-specific effects on the compositions of both the stool and the small bowel microbiomes in male vs. female rats. Furthermore, after cessation of antibiotics, both the stool and small bowel microbiomes remained different in males vs. females, and did not return to baseline profiles in either sex by the time of euthanasia. These findings suggest that exposure to antibiotics has sex-specific, and long-reaching, implications for the microbiome throughout the gastrointestinal tract.

When prescribing antibiotics, in addition to considering efficacy against the target organism(s), a physician will typically consider a patient’s age, weight, and prior antibiotic use to determine dosage (Patel et al., 2021). Sex is taken into consideration when there is a pregnancy, in part to address issues of concern with the fetus, and because the physiological changes associated with pregnancy may lead to alterations in absorption and distribution of pharmacological agents (Patel et al., 2021). Otherwise, sex is rarely considered when prescribing. Further, despite known sex-based differences in the drug activities of aspirin (Berger et al., 2006), beta blockers (Luzier et al., 1999), opioids (Berkley, 1997; Craft, 2003), selective serotonin reuptake inhibitors (Baca et al., 2004), tricyclic antidepressants (Baca et al., 2004), and typical antipsychotics (Seeman, 2004), relatively few studies have examined the effects of antibiotics with respect to sex.

A growing concern regarding antibiotic use is the potential effects on the gut microbiome, and studies of antibiotic exposure during childhood suggest that these effects may be sex-specific (Trasande et al., 2013; Bailey et al., 2014). Another concern regarding antibiotics is the potential for developing antibiotic resistance, and data from recent human gut metagenome studies suggest that the development of antibiotic resistance may be different in males and females. Further, sex-specific antibiotic resistome profiles have been identified, including a higher richness of antibiotic resistance genes in female subjects when compared to males (Sinha et al., 2019). These findings illustrate the importance of identifying sex-specific differences in the effects of antibiotics on the gut microbiome, as well as sex-specific differences the recovery of the gut microbiome following exposure to antibiotics.

In this study, we exposed male and female rats to an antibiotic cocktail for 8 days, and followed them for a further 13 day recovery period after antibiotics cessation. Beta-diversity analysis indicated that the stool microbiomes in male and female rats were very similar prior to antibiotic exposure, and remained similar in male and female control rats throughout the study. By day 8 of antibiotic exposure (AbxD8), exposed rats of both sexes exhibited a loss of microbial alpha diversity, consistent with an enrichment of specific populations following the loss of more susceptible taxa, and this was more pronounced in male rats.

In addition to this loss of diversity, by AbxD8 the composition of the stool microbiomes in male and female rats were significantly different from each other. Exposed male rats had significantly higher RA of the phyla Proteobacteria and Verrucomicrobia when compared to exposed females, whereas exposed females had a higher RA of phylum Firmicutes. Of note, both Proteobacteria and Verrucomicrobia are Gram-negative, whereas Firmicutes is Gram-positive. The outer membrane of Gram-negative bacteria functions as a barrier that can prevent antibiotics from penetrating the cell, and thus contributes to antibiotic resistance (Exner et al., 2017). In recent years there have been concerning increases in infections with multi-drug resistant Gram-negative bacteria (Exner et al., 2017), and the enrichment of Gram-negatives we identified in male rats after antibiotic exposure may be clinically relevant.

We also identified a higher RA of family Porphyromonadaceae (phylum Bacteroidetes) in the stool microbiome of male vs. female rats by AbxD8. Higher abundance of Porphyromonadaceae (among other taxa) was previously demonstrated in male mice and in female germ-free mice gavaged with conventional male microbiota (Fransen et al., 2017). Female recipients of male microbiota also had greater inflammation, weight loss, and DNA damage than those receiving female microbiota (Fransen et al., 2017). Coupled with the increased RA of Porphyromonadaceae previously identified in non-obese diabetic (NOD) mice lacking the innate immune system adapter MyD88 (Wen et al., 2008), this led the authors to suggest a potential role for Porphyromonadaceae in sex-based differences in innate immunity (Fransen et al., 2017).

In order to compare how the stool microbiome recovers in males vs. females after exposure to broad-spectrum antibiotics, we continued to monitor the rats for 13 days after antibiotics cessation, equivalent to more than 1 year of human life (Sengupta, 2013). At the end of this 13-day period (PAbxD13), neither the male nor the female stool microbiomes had recovered to baseline profiles, and remained significantly different from each other at all taxonomic levels. Male rats continued to exhibit decreased RA of phylum Actinobacteria when compared to female rats, and this was even more pronounced by PAbxD13 than on AbxD8. This is significant because, although Actinobacteria make up a small percentage of the gut microbiota, they are important in the maintenance of gut homeostasis (Binda et al., 2018). Genus *Bifidobacterium*, possibly the most significant gut Actinobacteria taxon, plays a key role in gut barrier maintenance by producing the short chain fatty acid acetate, which protects against enteric pathogens such as *Escherichia coli* and *Shigella* (Fukuda et al., 2012; Hardy et al., 2013; Binda et al., 2018). Phylum Bacteroidetes also demonstrated a sex-specific recovery in the stool microbiome. The RA of Bacteroidetes was significantly reduced following antibiotic exposure in both sexes, but by PAbxD13, had returned to baseline levels in females only. In contrast, the RA of Bacteroidetes in male rats on PAbxD13 was increased compared to baseline, and was significantly higher than in female rats. Many Bacteroidetes species can be beneficial to the host, and function as commensal bacteria in the gut (Wexler, 2007). Further, Bacteroidetes species have the greatest number of antibiotic resistance mechanisms and the highest rates of resistance among anaerobic pathogens (Wexler, 2007). Our finding of increased prevalence of Bacteroidetes in male rats following recovery from very high doses of broad-spectrum antibiotics may therefore have clinical implications, such as an increased potential for bacteremia, although further work will be needed to confirm this.

At the family level, on PAbxD13, the stool microbiome of female rats had a higher RA of Enterobacteriaceae (phylum Proteobacteria) when compared to male rats. This difference was not present at baseline, and during antibiotic exposure the RA of Enterobacteriaceae was higher in male rats. Enterobacteriaceae is a family of Gram-negative bacteria that contains several pathogens, including *E. coli*, *Klebsiella pneumoniae*, and *Salmonella*. An unknown genus from Enterobacteriaceae also had higher RA in female rats when compared to males after cessation of antibiotics. Enterobacteriaceae are known to be resistant to certain antibiotics due to the acquisition of plasmids containing genes encoding extended spectrum beta-lactamases (Paterson, 2006). Our finding of higher RA of Enterobacteriaceae in female rats as compared to males at the end of the antibiotic recovery period is interesting, but its clinical significance remains to be determined. Lastly, on PAbxD13, the stool microbiome of exposed male rats exhibited higher RA of the genera *Parabacteroides* when compared to females. *Parabacteroides* is a relatively new genus reclassified from genus *Bacteroides* and is particularly noted for its antibiotic resistance (Ezeji et al., 2021).

In order to determine whether these sex-specific antibiotic-induced changes in the stool microbiome reflected changes throughout the gastrointestinal tract, the small bowel microbiome in male and female rats was compared using samples harvested at euthanasia. In control rats, there were few differences between the small bowel microbiota of males and females at euthanasia, primarily a lower RA of genus *Akkermansia* (phylum Verrucomicrobia) in males that mirrored findings in stool, and a higher RA of *L. reuteri* in males that was unique to the small bowel. Although the significance of this is unknown, we note that when given as probiotics in drinking water, *L. reuteri* strains have sex-specific effects on cytokine and insulin levels (Lee et al., 2016). In contrast, the small bowel microbiome profiles of exposed rats were significantly different from controls. Moreover, many of the sex-specific differences in the stool microbiota following antibiotics exposure were also seen in the small bowel, suggesting that they were reflective of changes throughout the gastrointestinal tract. These included lower RA of the phyla Actinobacteria, Verrucomicrobia, and Proteobacteria in exposed males when compared to exposed females. Interestingly, while the phyla Firmicutes and Bacteroidetes were also affected by antibiotics exposure in both stool and the small bowel, the overall RA of Firmicutes was higher in the small bowel of exposed males when compared to exposed females, and the overall RA of Bacteroidetes was lower, the opposite of findings in stool. Within these phyla, genera including *Parabacteroides*, *Staphylococcus*, *Roseburia*, *Ruminococcus* and *Bifidobacterium* exhibited sex-specific changes in both the stool and small bowel microbiomes, although sex-specific differences in others (e.g., *Sutterella*) only reached significance in the small bowel.

The taxa that were different between the small bowel and stool microbiota in control males were also different between the small bowel and stool microbiota in exposed males, suggesting that in males, microbial profiles return to profiles closer to baseline (normal) following exposure to antibiotics. In contrast, the taxa that were different between small bowel and stool microbiota in control females were not the same as those that were different between small bowel and stool microbiota in exposed females. These findings may suggest that in females, different taxa colonize certain niches following exposure to antibiotics, such that profiles do not return to baseline/normal, but rather that new profiles emerge. However, further work is needed to confirm this, and to determine whether this represents an advantageous or a deleterious change. We note that the antibiotic cocktail used in this study represents significantly higher antibiotic doses than would be received in any typical course of antibiotic therapy, but the differential effects in males vs. females are nonetheless striking.

Previous studies in mice also found sex-specific differences in the effects of antibiotics on the stool microbiome (Gao et al., 2019; Harrison et al., 2019). However, comparisons between those studies and ours were complicated by the fact that, interestingly, there were far more baseline differences between male and female stool microbiota in mice than we found in rats. For example, Gao et al. found significantly lower RA of phyla Firmicutes and Deferribacteres, and higher RA of phyla Bacteroidetes and Proteobacteria, in female C57Bl/6 mice at baseline (6 weeks of age; Gao et al., 2019), whereas we only found higher RA of phylum Verrucomicrobia in female vs. male rats at baseline (8 weeks of age). At the genus level, RA of *Lactobacillus*, *Prevotella*, and *Sutterella* were higher, and RA of nine genera were lower, in female vs. male mice (Gao et al., 2019), whereas we only found higher RA of 4 genera, *Akkermansia*, *Lactococcus*, *Coprococcus* and an unknown genus from order RF32, in female vs. male rats. Although the reasons for the greater degree of microbial disparity between sexes in mice vs. rats are unknown, previous studies have noted differences between the mouse and rat stool microbiomes, including higher abundances of Proteobacteria and lower Verrucomicrobia in rats (Nagpal et al., 2018). There were also differences in the antibiotics administered and duration of treatment, as Gao et al. administered either 0.5 g/L vancomycin or a combination of 0.2 g/L ciprofloxacin and 1 g/L metronidazole to separate groups for 2 weeks (Gao et al., 2019). Despite this, there were some consistencies with our findings, including higher RA of *Sutterella* in male mice after vancomycin treatment, and higher RA of *Enterococcus* in female mice after ciprofloxacin/metronidazole treatment (Gao et al., 2019). Although Harrison et al. studied *Rag2−/−* mice on a 129/SvEv background rather than wild-type animals, and were concurrently treating with antibiotics (0.2 g/L ciprofloxacin and 0.5 g/L metronidazole) and injecting with naive T cells to induce inflammation (as a mouse model of colitis), we note that they also found stool microbiome differences between male and female mice, both at baseline and during Abx treatment (Harrison et al., 2019). These included a significant expansion of family Lactobacillaceae that was greater in males than in females, and an expansion of Enterococcaceae that was greater in females (Harrison et al., 2019), which is consistent with our findings of higher RA of *Lactobacillus* in male vs. female rats, and higher RA of *Enterococcus* in females, after 8 days of Abx. Further, utilizing our rat model enabled us to also examine the effects of antibiotics on the small bowel microbiome, and to compare the results to our findings in stool.

We also examined potential microbial functional differences in antibiotic-exposed male vs. female rats, and found that even on PAbxD13, there were still significant differences in predicted stool microbial metabolic pathways, including enrichments in different carbohydrate biosynthesis and metabolism pathways as well as fatty acid biosynthesis in exposed males vs. females. The latter finding is consistent with Harrison et al., who also found enrichment for fatty acid and lipid biosynthesis pathways in exposed male vs. female mice (Harrison et al., 2019). In addition, two stool microbial pathways predicted to be enriched in our exposed female rats, glycine, serine and threonine metabolism and terpenoid backbone metabolism, were also predicted to be enriched in the small bowel, suggesting that these may represent persistent microbial functional differences long after antibiotic cessation.

An interesting finding was that while both male and female rats showed significant weight loss during antibiotic exposure, exposed females fully recovered their weights following antibiotic cessation, whereas exposed males still had lower weights at the end of the study when compared to controls. Examination of BCR ratios revealed similar increases and subsequent decreases in BCR in male and female rats during antibiotic exposure and recovery, respectively, suggesting that the lack of weight recovery after cessation of antibiotics in male rats was unlikely to be driven by dehydration. We note that on AbxD8, the RA of phylum Verrucomicrobia, primarily represented by genus *Akkermansia*, was increased in male rats when compared to baseline, and that this had a strong inverse association with weight, which is interesting as increased levels of *Akkermansia muciniphila* have been associated weight loss in human subjects with obesity (Xu et al., 2020). Moreover, Fransen et al. found that germ-free female mice that received microbiota from male donors lost significantly more weight than those who received microbiota from female animals, and that they exhibited persistent lower weights for the 4-week duration of their experiment (Fransen et al., 2017), supporting a sex-specific relationship between gut microbiota and weight.

This study has some limitations. First, a multi-drug broad-spectrum antibiotic cocktail was used. This was intentional as we sought to affect a wide range of microbes and induce a readily observable response. Our next steps will be to focus on specific antibiotics to determine the sex-specific effects individually. Second, the study only included one post-exposure recovery timepoint at 13 days following cessation of antibiotics. As noted, this corresponds to more than 1 year of human life. Studies of adult human subjects exposed to two 5-day “pulses” of ciprofloxacin 6 months apart found that most, but not all, taxa recovered to pre-exposure RAs within 2 weeks of the first exposure, but found less complete recovery after the second exposure (Dethlefsen et al., 2008; Dethlefsen and Relman, 2011), suggesting that exposure even to therapeutic doses of antibiotics may have lasting effects on the gut microbiome. Further studies following rats for longer recovery periods will be required to determine if the stool and/or small bowel microbiomes in either sex eventually recover to pre-antibiotic profiles.

In conclusion, our data suggest that a broad-spectrum multi-drug antibiotic cocktail has significant sex-specific effects on both the small bowel and the stool microbiomes of male and female rats. After a 13-day recovery period, the stool microbiomes in both sexes remain significantly different from baseline, and the small bowel microbiomes remain significantly different from controls. Importantly, there may be greater effects on the small bowel microbiome *profiles* in exposed females than in exposed males, although there are greater effects on small bowel microbial *diversity* in males. These findings may have important implications for the way antibiotics are prescribed. Future studies with single antibiotics, different combinations of antibiotics, and different exposure/recovery timelines will allow us to further understand the implications for clinical practice.

## Data Availability Statement

The datasets presented in this study can be found in online repositories. The datasets can be found at: https://www.ncbi.nlm.nih.gov/bioproject under BioProject ID PRJNA802267.

## Ethics Statement

The animal study protocol was reviewed and approved by the Institutional Animal Care and Use Committee of Cedars-Sinai Medical Center, Los Angeles, CA, United States.

## Author Contributions

RM: conceptualization. GP, GL, MLP, and WM: investigation. GL and MP: formal analysis. GB and RM: project administration. WM and SW: supervision. GP, AF, MLP, GL, GB, and RM: writing original draft. GP, GL, GB, MP, and RM: writing—review and editing. All authors contributed to the article and approved the submitted version.

## Funding

This study was supported in part by grants from Monica Lester Charitable Trust and the Elias, Genevieve, and Georgianna Atol Charitable Trust to RM.

## Conflict of Interest

The authors declare that the research was conducted in the absence of any commercial or financial relationships that could be construed as a potential conflict of interest.

## Publisher’s Note

All claims expressed in this article are solely those of the authors and do not necessarily represent those of their affiliated organizations, or those of the publisher, the editors and the reviewers. Any product that may be evaluated in this article, or claim that may be made by its manufacturer, is not guaranteed or endorsed by the publisher.

## Footnotes

^1^16S Metagenomic Sequencing Library Preparation. Available at: https://support.illumina.com/documents/documentation/chemistry_documentation/16s/16s-metagenomic-library-prep-guide-15044223-b.pdf
