# Supplementary material for: The Response of the Rodent Gut Microbiome to Broad-Spectrum Antibiotics Is Different in Males and Females
Source: Front Microbiol. 2022 Jun 9;13:897283. doi: 10.3389/fmicb.2022.897283 (PMC9218673; doi:10.3389/fmicb.2022.897283)
Supplement: Supplementary file 1 [file Data_Sheet_1.docx]

Supplementary Material

# Supplementary Figures and Tables

## Supplementary Figures

**
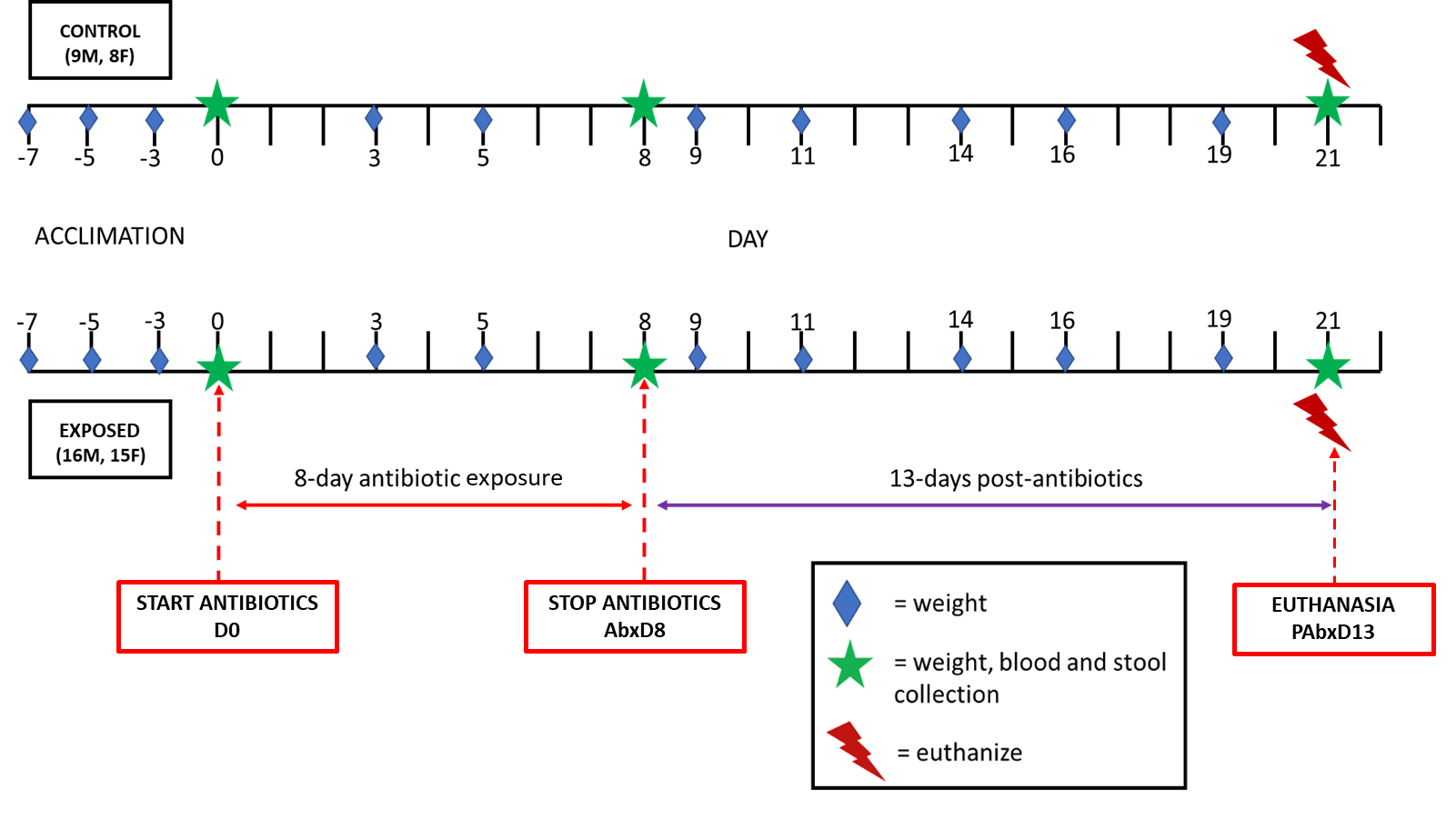
**

**Supplementary Figure 1.** Study Design. Male and female rats were randomized into two groups and either exposed to a multi-drug antibiotic cocktail for 8 days, or remained unexposed as controls. After cessation of antibiotics, rats were monitored for an additional 13-day recovery period, and then euthanized. Small bowel samples were harvested at euthanasia. Rats were weighed and blood and stool samples were collected at the indicated timepoints.


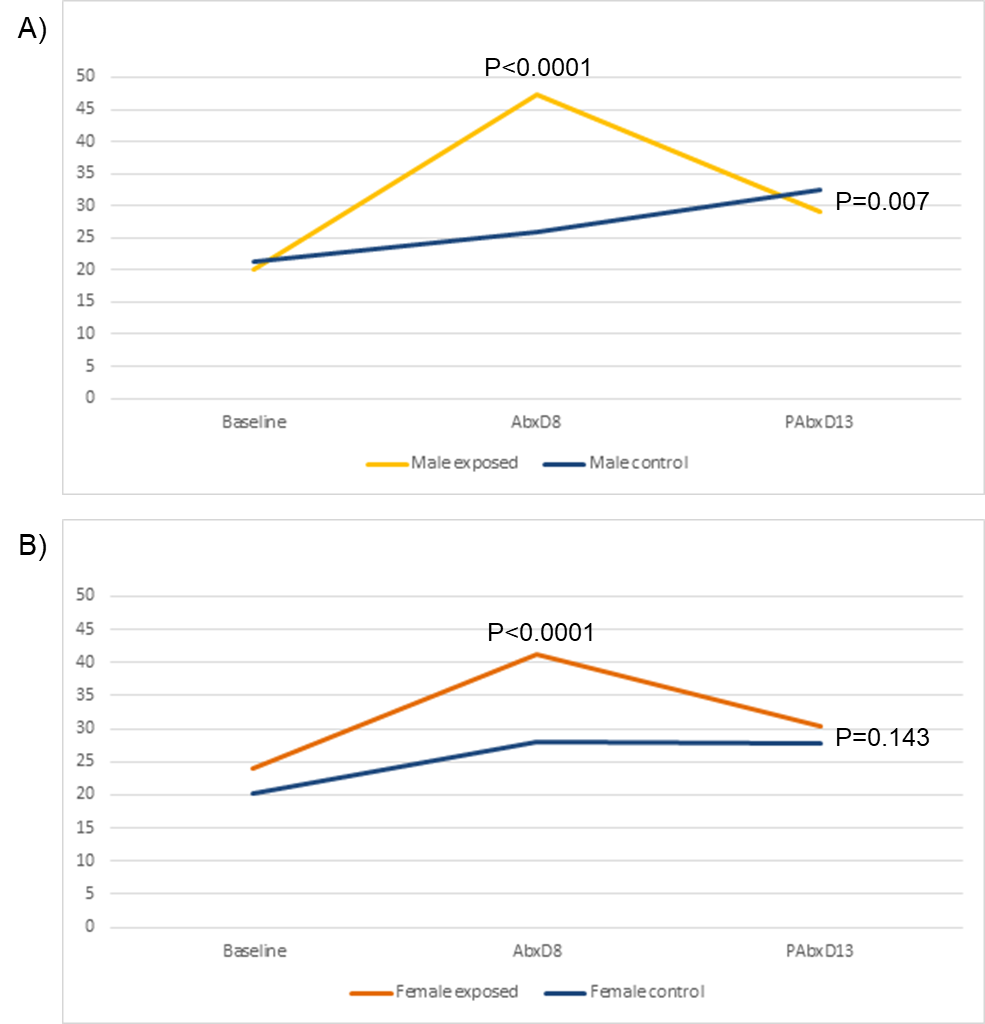


**Supplementary Figure 2.** Blood urea nitrogen (BUN)-to-creatinine ratios (BCRs). A) BCRs in male exposed and control rats at baseline, after 8 days of antibiotic exposure (AbxD8), and 13 days after cessation of antibiotics (PAbxD13). B) BCRs in female exposed and control rats at baseline, on AbxD8, and on PAbxD13.

**
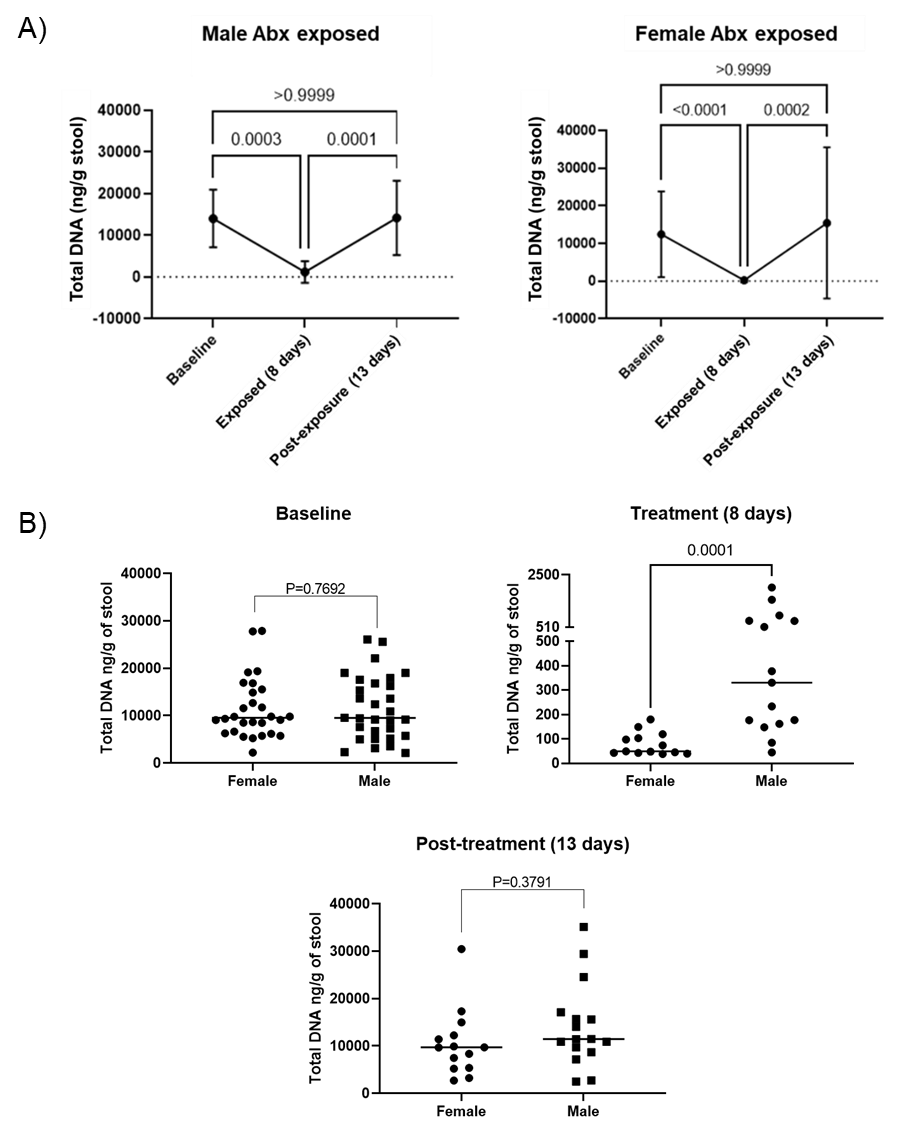
**

**Supplementary Figure 3.** Total stool DNA levels. A) Total stool DNA in male and female rats at baseline, after 8 days of antibiotic exposure (AbxD8), and 13 days after cessation of antibiotics (PAbxD13). B) Comparison of total stool DNA levels in male and female exposed rats at baseline, on AbxD8, and on PAbxD13.

**
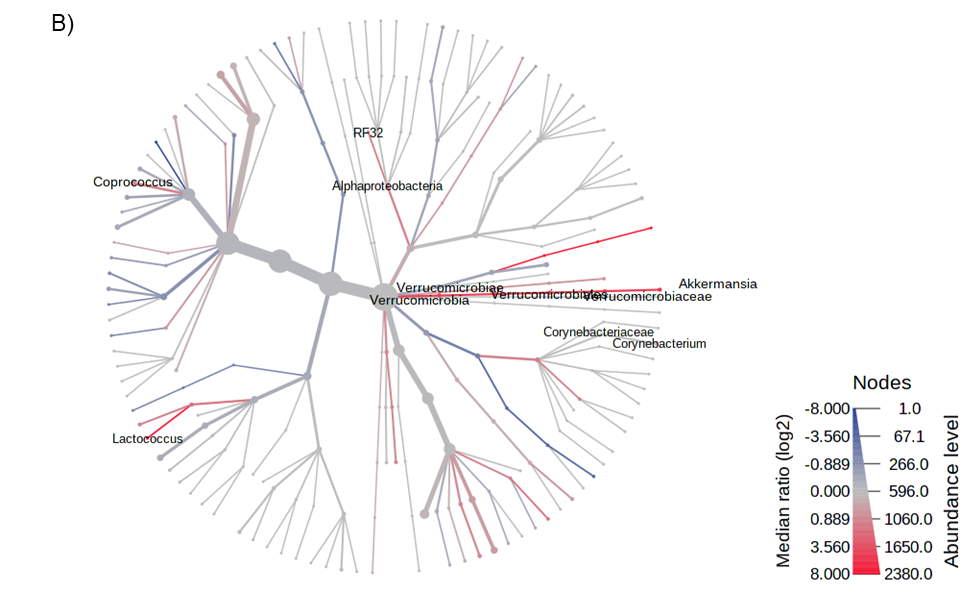
**

**Supplementary Figure 4.** Heat tree of the stool microbiome in male vs. female exposed rats at baseline. Taxa with lower relative abundance (RA) in males vs. females are shown in red. Taxa with higher RA in males vs. females are shown in blue. Nodes represent taxonomic levels, and greater line thickness denotes higher RA.

**
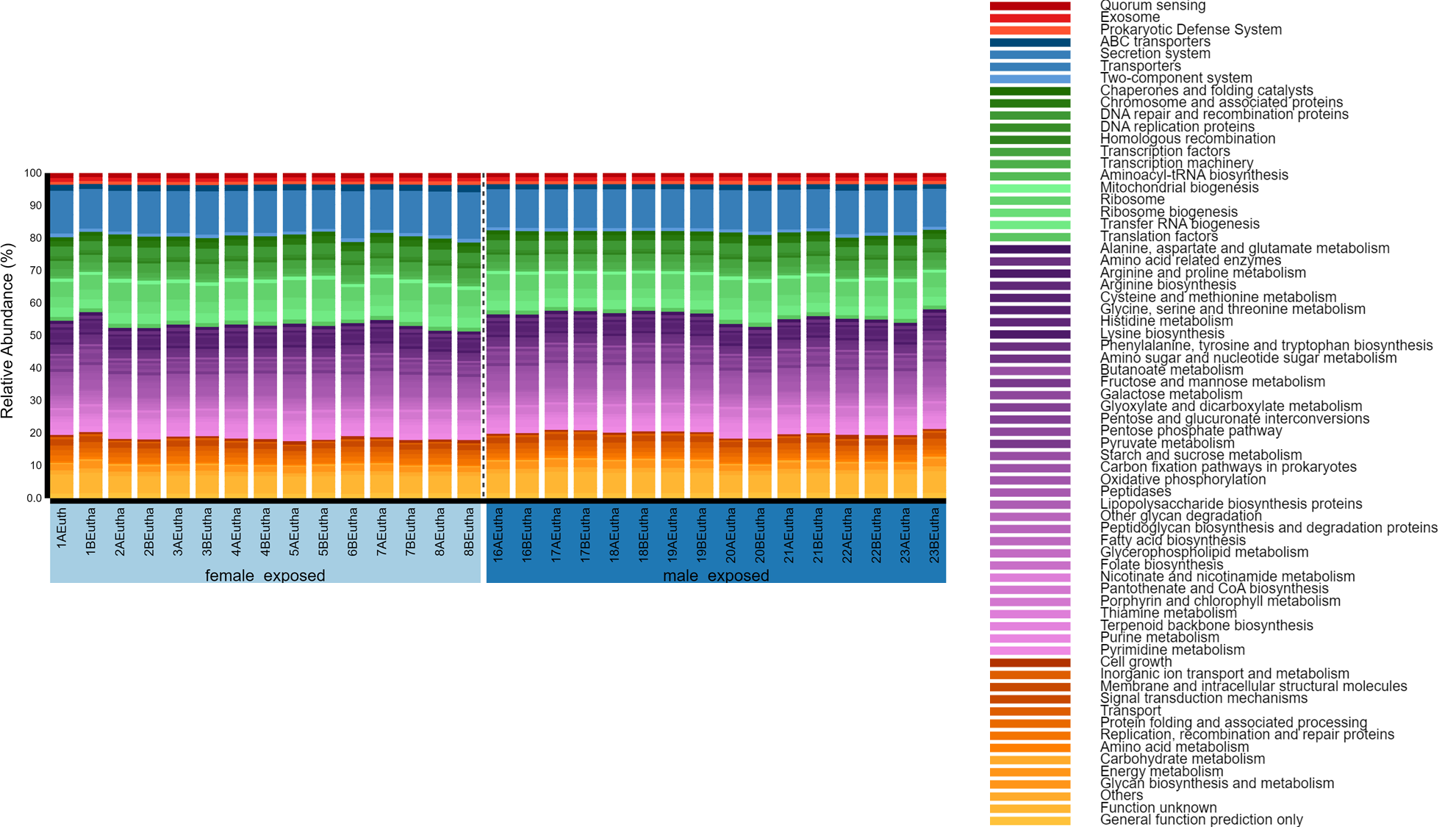
**

**Supplementary Figure 5.** Differences in predicted stool microbial functional metabolic pathways in exposed male and female rats on PAbxD13.


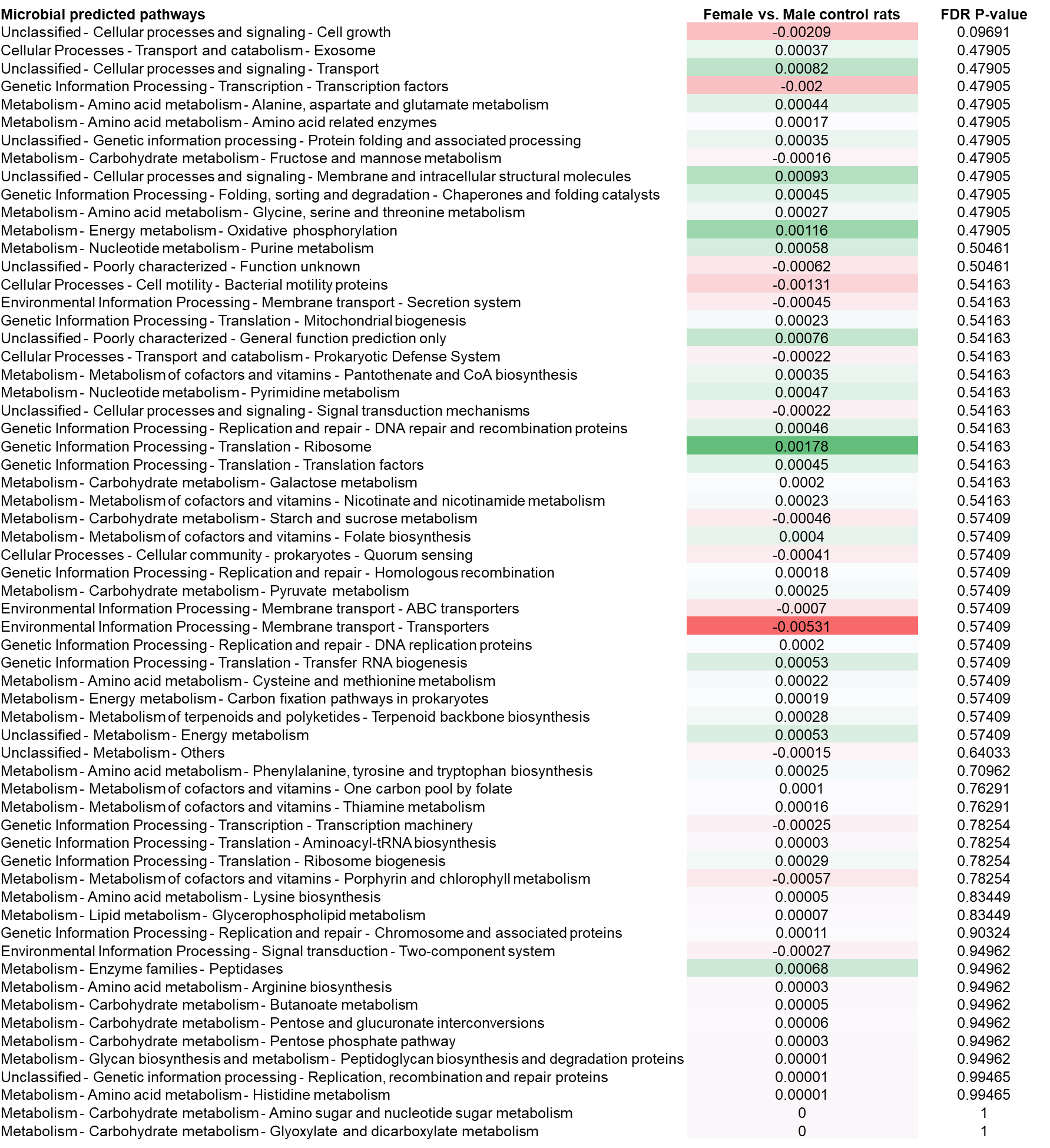


**Supplementary Figure 6.** Differences in predicted stool microbial functional metabolic pathways in control male and control female rats on PAbxD13. Pathways shown in red are predicted to be enriched in control males, and pathways shown in green are predicted to be enriched in control females. None of the differences shown reached significance after FDR correction**.**


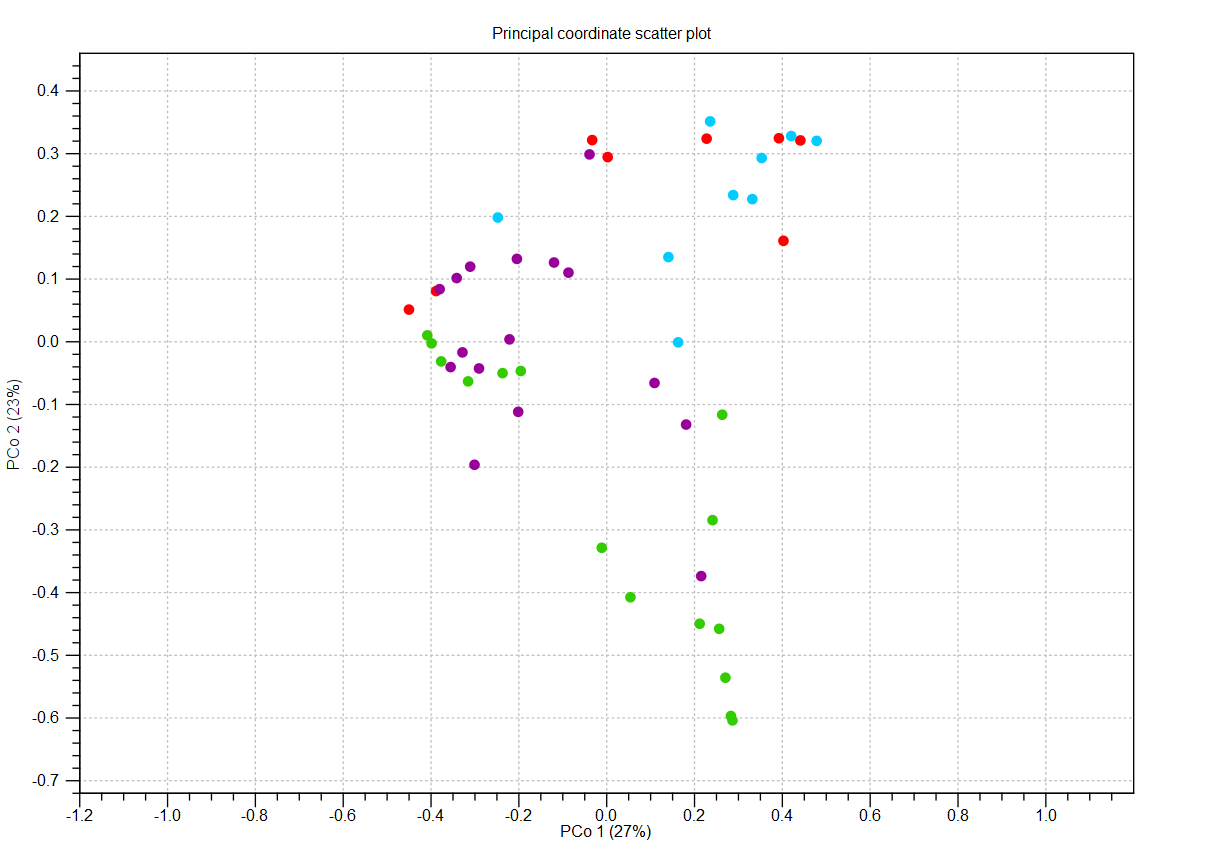


A)


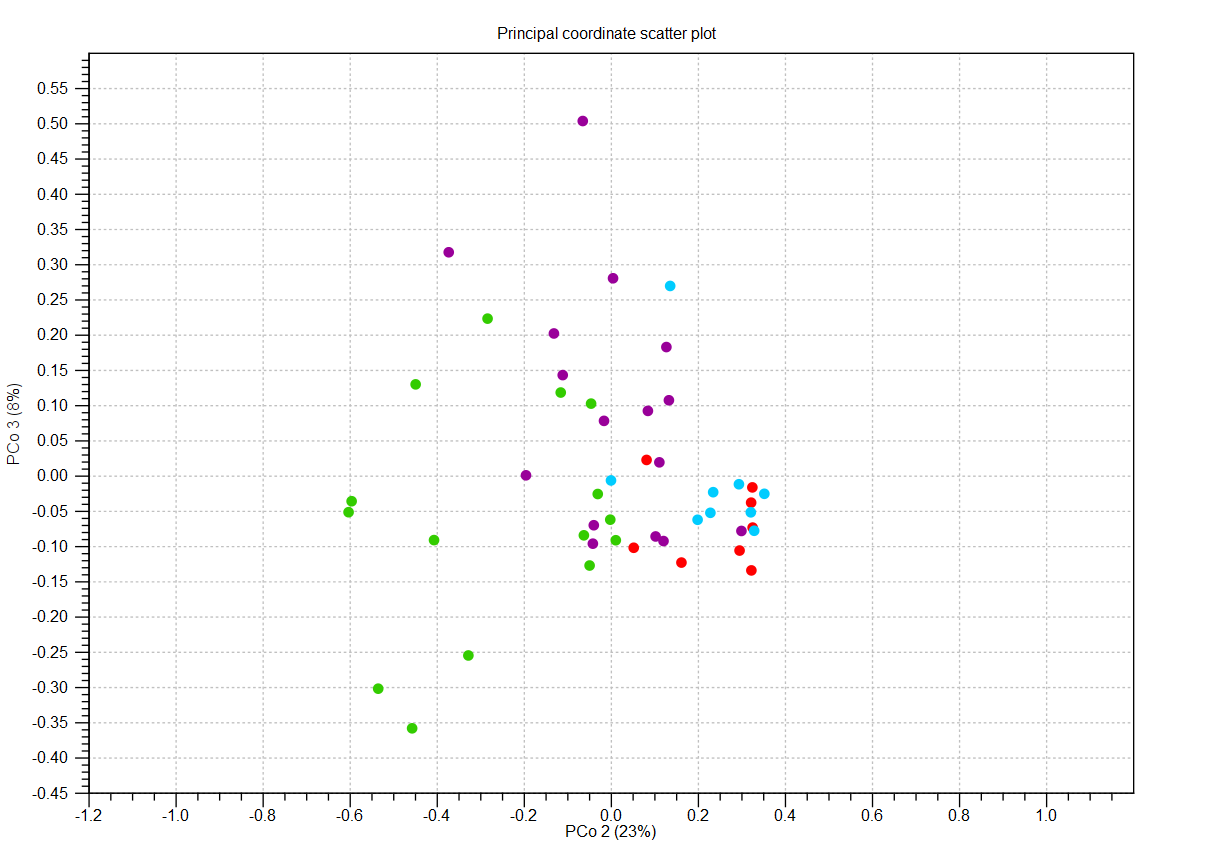


B)


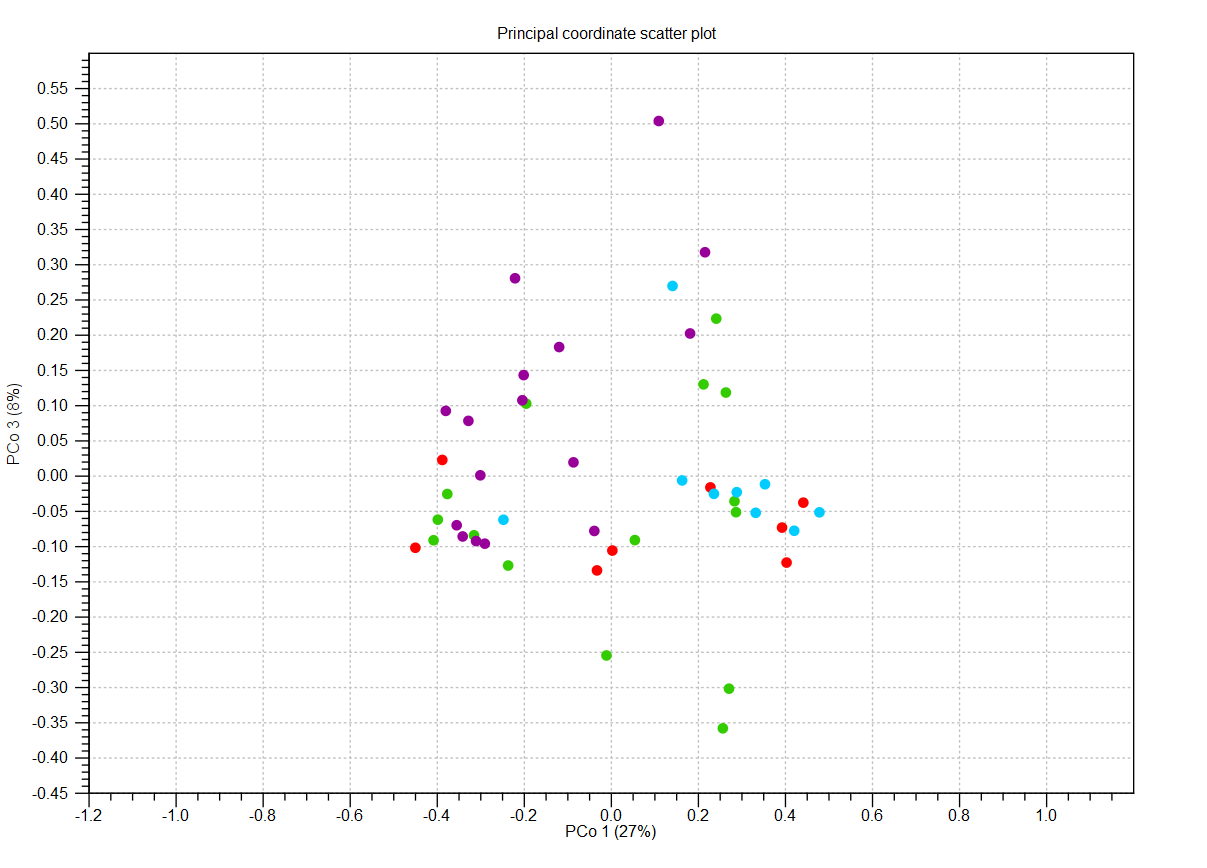
**Supplementary Figure 7.** Two-dimensional principal component analysis (PCA) plots of small bowel beta diversity in male and female rats on PAbxD13. A) PC1 vs PC2, B) PC2 vs PC3, and C) PC1 vs PC3. Control males - red, control females - blue, exposed males - purple, exposed females – green. Male vs female controls: PERMANOVA P=1. Exposed females vs exposed males PERMANOVA p=0.01026. Exposed females vs female controls: PERMANOVA p=0.0022. Exposed males vs male controls: PERMANOVA p=0.00006).

C)


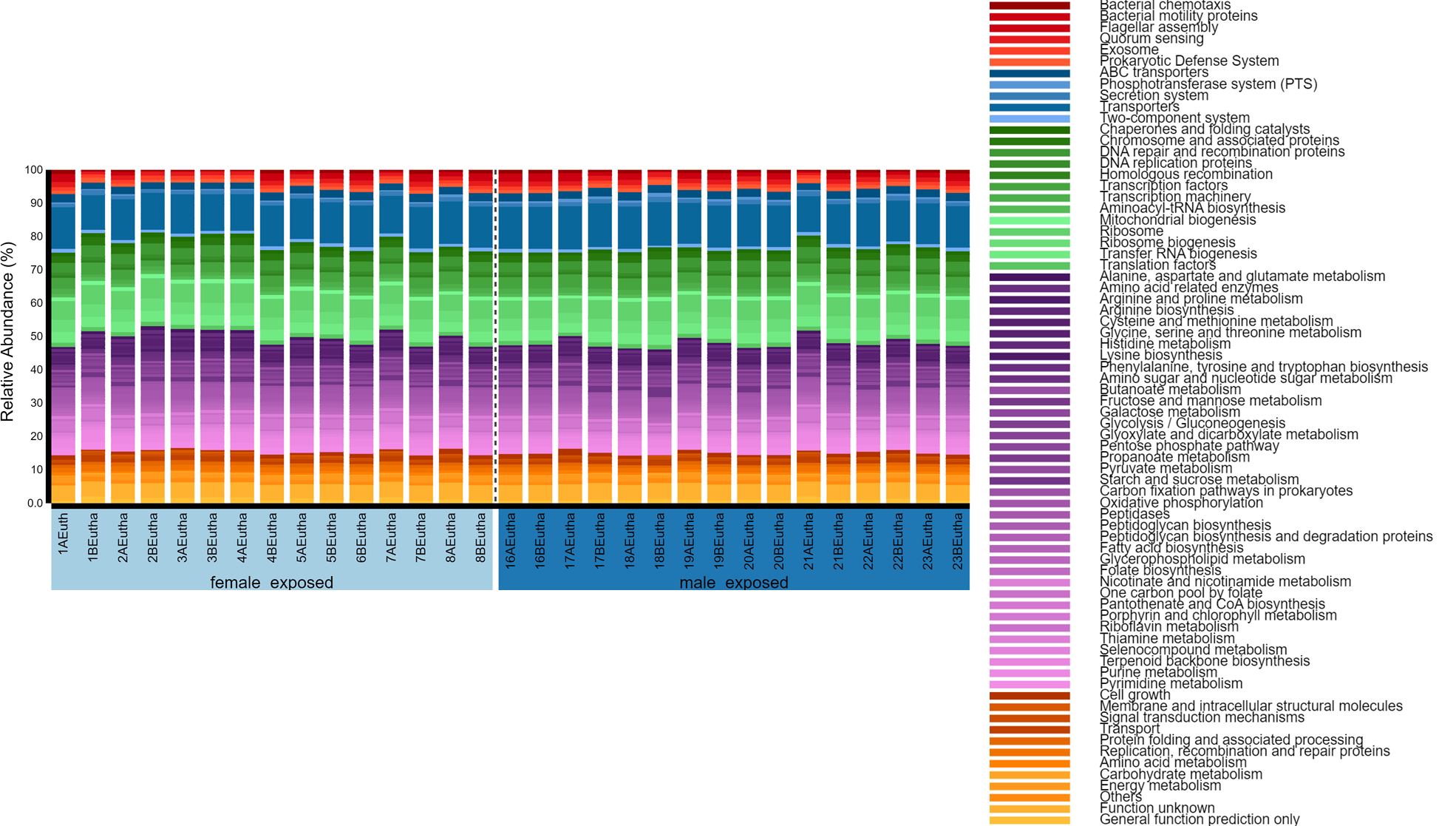


**Supplementary Figure 8.** Differences in predicted small bowel microbial functional metabolic pathways in exposed male and female rats on PAbxD13.

**
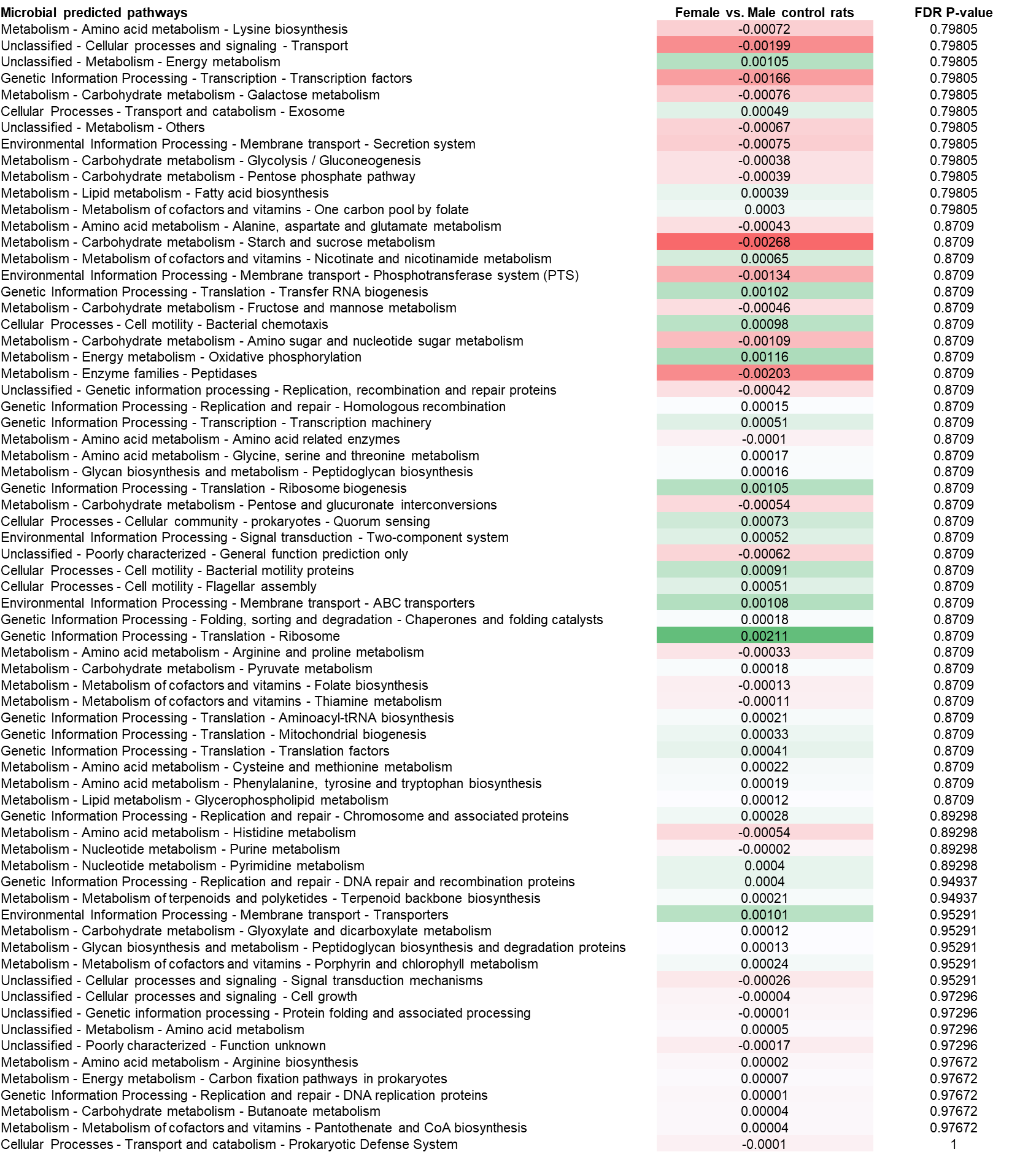
**

**Supplementary Figure 9.** Differences in predicted small bowel microbial functional metabolic pathways in control male and control female rats on PAbxD13. Pathways shown in red are predicted to be enriched in control males, and pathways shown in green are predicted to be enriched in control females. None of the differences shown reached significance after FDR correction**.**


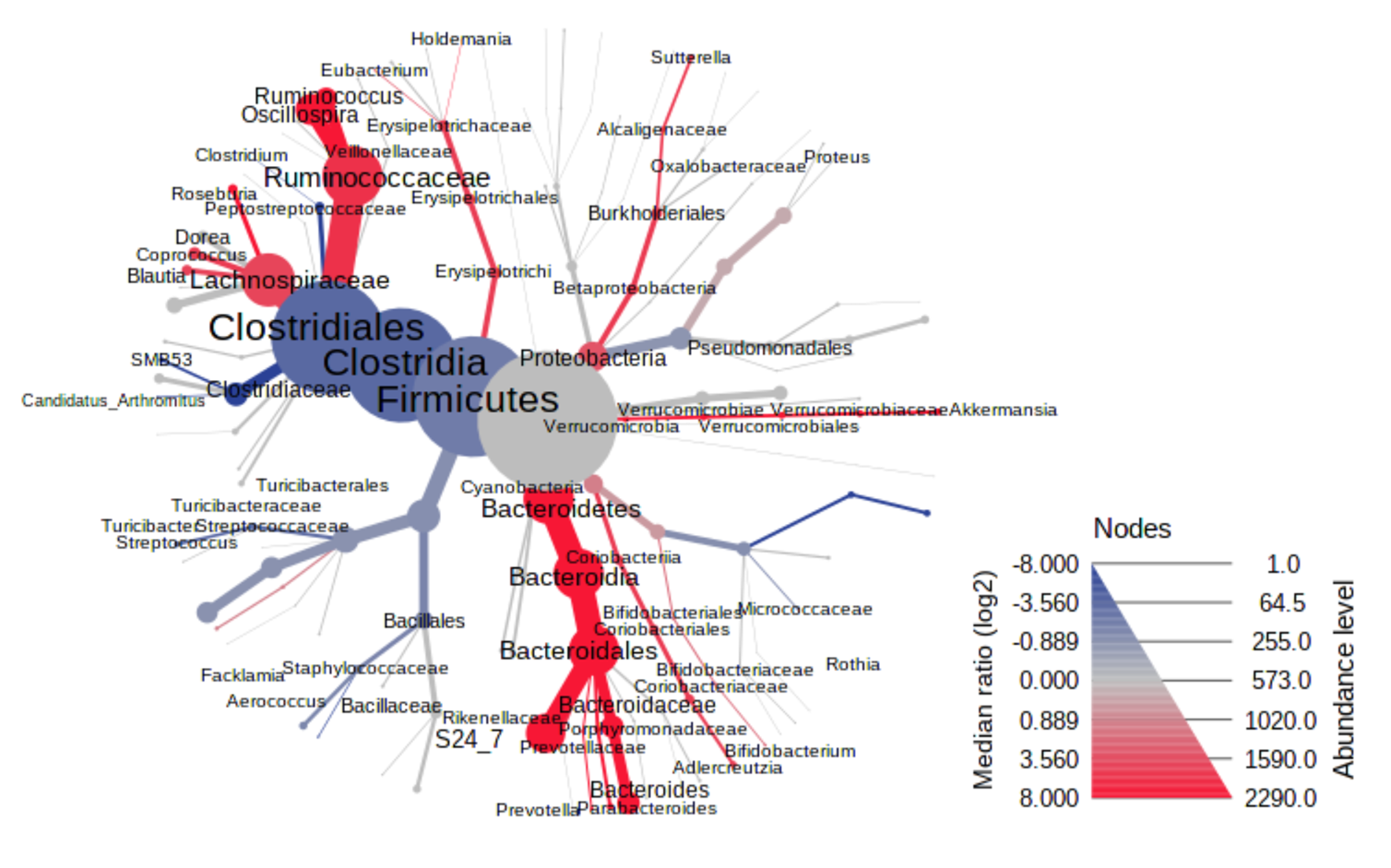


**Supplementary Figure 10.** Heat tree of the stool vs. small bowel microbiomes in male exposed rats on PAbxD13. Taxa with lower RA in the small bowel vs. stool are shown in red. Taxa with higher RA in the small bowel when compared to stool are shown in blue. Nodes represent taxonomic levels. Greater line thicknesses and greater font sizes denote higher RA.

## Supplementary Table 1. Rat weights and blood urea nitrogen (BUN)-to-creatinine ratios (BCRs) during the acclimation, antibiotic exposure and post-antibiotic recovery periods.

|  | **Days Prior to ABX Exposure** | | | | **ABX Exposure Period Days** | | | | | **Post-ABX Recovery Period Days** | | | | | | | |
| --- | --- | --- | --- | --- | --- | --- | --- | --- | --- | --- | --- | --- | --- | --- | --- | --- | --- |
|  | **-7** | **-5** | **-3** | | **0** | **3** | **5** | **8 (AbxD8)** | | **9** | **11** | **14** | **16** | **19** | **21 (PAbxD13)** | |  |
|  | **Weight (g)** | **Weight (g)** | **Weight (g)** | **BCR** | **Weight (g)** | **Weight (g)** | **Weight (g)** | **Weight (g)** | **BCR** | **Weight (g)** | **Weight (g)** | **Weight (g)** | **Weight (g)** | **Weight (g)** | **Weight (g)** | **BCR** |  |
| **Male**  **Control** | 269.8  ±9.5 | 276.8  ±8.4 | 280.9  ±11.7 | 21.0  ±3.3 | 292.7  ±11.6 | 294.2  ±12.9 | 301.7  ±13.3 | 314.3  ±14.7 | 26.0  ±8.4 | 313.3  ±14.7 | 317.5  ±15.2 | 325.6  ±15.6 | 327.6  ±16.6 | 332.1  ±16.4 | 337.5  ±17.2 | 32.4  ±3.3 |  |
| **Male**  **Exposed** | 269.4  ±9.3 | 274.1  ±10.2 | 281.8  ±10.8 | 20.1  ±3.2 | 292.9  ±12.1 | 267.5  ±12.2 | 249.2  ±13.2 | 255.6  ±39.3 | 47.3  ±9.3 | 278.0  ±25.5 | 289.1  ±21.0 | 305.0  ±18.5 | 299.4  ±19.6 | 309.0  ±25.3 | 323.1  ±19.0 | 29.0  ±3.1 |  |
| **Female**  **Control** | 194.7  ±4.7 | 194.5  ±5.9 | 198.9  ±13.0 | 20.3  ±3.9 | 202.7  ±6.4 | 204.2  ±5.8 | 205.2  ±7.1 | 214.9  ±6.5 | 28.1  ±3.4 | 211.6  ±7.6 | 215.2  ±7.9 | 220.4  ±8.7 | 222.4  ±8.8 | 225.2  ±7.7 | 227.2  ±7.2 | 25.8  ±9.1 |  |
| **Female**  **Exposed** | 200.4  ±6.4 | 200.9  ±5.9 | 199.4  ±5.3 | 24.0  ±2.3 | 202.1  ±5.2 | 186.4  ±6.5 | 180.3  ±13.0 | 212.5  ±6.8 | 41.1  ±3.7 | 215.5  ±7.0 | 215.3  ±12.0 | 224.7  ±6.2 | 219.5  ±10.2 | 221.2  ±23.0 | 221.1  ±6.9 | 30.3  ±3.7 |  |

ABX – antibiotics; BCR - blood urea nitrogen (BUN)-to-creatinine ratio; g – grams
